# Cardiovascular outcomes of empagliflozin-GLP-1RA combination therapy in type 2 diabetes: EMPRISE study

**DOI:** 10.1186/s12933-026-03287-w

**Published:** 2026-07-12

**Authors:** Phyo T. Htoo, Hanseul Cho, Julie M. Paik, Katsiaryna Bykov, Brendan M. Everett, Robert J. Glynn, Christina Shay, Niklas Schmedt, Deborah J. Wexler, Elisabetta Patorno

**Affiliations:** 1 Division of Pharmacoepidemiology and Pharmacoeconomics, Department of Medicine, Brigham and Women’s Hospital and Harvard Medical School, Boston, MA USA; 2 Division of General Internal Medicine and Health Services Research, David Geffen School of Medicine, University of California Los Angeles, 1100 Glendon Ave., Los Angeles, CA 90024 USA; 3 Division of Nephrology, David Geffen School of Medicine at UCLA, Los Angeles, CA USA; 4 Divisions of Cardiovascular and Preventive Medicine, Department of Medicine, Brigham and Women’s Hospital and Harvard Medical School, Boston, MA USA; 5 TA CRM, Boehringer Ingelheim International GmbH, Ingelheim, Germany; 6 Diabetes Unit, Massachusetts General Hospital and Harvard Medical School, Boston, MA USA

**Keywords:** Empagliflozin, Glucagon-like peptide-1 receptor agonist, Sodium-glucose cotransporter-2 inhibitor, Type 2 diabetes mellitus, Cardiovascular disease, Heart failure

## Abstract

**Background:**

Evidence on the cardiovascular benefits of combining empagliflozin with glucagon-like peptide-1 receptor agonists (GLP-1RA) is limited, derived primarily from indirect comparisons or subgroup analyses of cardiovascular outcome trials.

**Methods:**

We conducted a cohort study emulating two comparative effectiveness trials using US healthcare databases (2013–2022), including federal Medicare and commercial claims data (Optum Clinformatics and MarketScan). Among adults with type 2 diabetes in routine care, we compared cardiovascular outcomes in patients initiating: (i) empagliflozin first, followed by augmentation with either GLP-1RA or dipeptidyl peptidase-4 inhibitors (DPP-4i), and (ii) GLP-1RA first, followed by augmentation with either empagliflozin or sulfonylureas. Primary outcomes were modified major adverse cardiovascular events (MACE; composite of myocardial infarction, stroke, and all-cause mortality) and a composite of hospitalization for heart failure (HHF) or all-cause mortality. We estimated hazard ratios (HR) and rate differences (RD) per 1,000 person-years with 95% confidence intervals (CI), adjusting for over 130 baseline covariates using propensity score matching weights.

**Results:**

Compared with empagliflozin-DPP-4i initiators, empagliflozin-GLP-1RA was associated with lower risk of MACE [HR: 0.81 (95% CI: 0.67, 0.97); RD: -3.7 (-7.1, -0.2)] and lower HHF-mortality [HR: 0.61 (0.48, 0.77); RD: -6.6 (-9.3, -3.9)]. Compared with the GLP-1RA-sulfonylurea combination, the GLP-1RA-empagliflozin combination had lower risks of MACE [HR: 0.68 (0.56, 0.81); RD: -5.3 (-7.9, -2.6)] and HHF-mortality [HR: 0.62 (0.51, 0.76); RD: -3.7 (-6.1, -1.8)].

**Conclusions:**

In US clinical practice, adding GLP-1RA to empagliflozin was associated with lower risks of MACE and HHF-mortality compared with adding DPP-4i. Among GLP-1RA initiators, adding empagliflozin was associated with lower cardiovascular risk than adding sulfonylureas, supporting potential additive cardiovascular benefits of GLP-1RA-empagliflozin combination therapy.

**Supplementary Information:**

The online version contains supplementary material available at https://doi.org/10.1186/s12933-026-03287-w.

**Research Insights**

**What is currently known about this topic?**

- GLP-1RAs reduce MACE and improve kidney outcomes in patients with T2D at high cardiovascular risk.
- Evidence on empagliflozin-GLP-1RA combination benefits derives mainly from indirect comparisons or subgroup analyses.
- SGLT2is show strong benefits for heart failure and kidney outcomes, while GLP-1RAs reduce stroke risk.

**What is the key research question?**

Is the combined use of empagliflozin and GLP-1RA associated with lower cardiovascular risk compared with alternative combination therapies in patients with type 2 diabetes?

**What is new?**

- GLP-1RA-Empagliflozin group had 32% lower risk of MACE compared with GLP-1RA-Sulfonylurea group.
- Empagliflozin-GLP-1RA showed 39% lower risk of heart failure hospitalization-mortality compared with Empagliflozin-DPP-4i group.
- Benefits were consistent across two treatment sequencing strategies (Empagliflozin-first and GLP-1RA-first).

**How might this study influence clinical practice?**

Real-world evidence is consistent with potential additive cardiovascular benefits of empagliflozin-GLP-1RA combination therapy, which may inform treatment intensification strategies for patients with type 2 diabetes.

## Introduction

Cardioprotective glucose-lowering medications have reshaped the management of type 2 diabetes [1, 2]. Glucagon-like peptide-1 receptor agonists (GLP-1RA) and sodium-glucose cotransporter 2 inhibitors (SGLT2i) have each shown benefits in reducing the risk of atherosclerotic cardiovascular disease (ASCVD), heart failure (HF), and kidney disease progression [3–7]. Among SGLT2is, empagliflozin has emerged as the most widely used agent in the U.S., making it a key focus in current clinical practice and research [8, 9]. Given their complementary mechanisms of action and beneficial effects beyond glycemic control, combining GLP-1RA and SGLT2i—particularly empagliflozin—may offer additive cardiovascular protection [1, 2, 8, 9]. The American Diabetes Association Standards of Care in Diabetes support the combined use of these agents in patients at high cardiovascular risk [1, 2].

Despite such recommendations, current evidence from systematic reviews and meta-analyses of cardiovascular outcome trials remains limited by the small number of patients who initiated and continued both therapies, as no randomized clinical trials (RCTs) have directly compared the cardiovascular benefits of combination therapy versus either treatment alone [10–15]. In addition, trial participants are typically younger and at higher cardiovascular risk than patients treated in routine clinical practice.

Non-randomized evidence on combination therapies is also limited by several factors, including narrow patient populations (e.g., individuals with myocardial infarction (MI)) who may not represent the broader type 2 diabetes population [16], earlier study periods when prescribing patterns and medication availability differed [17], or healthcare system differences (e.g., United Kingdom vs. United States), reducing the generalizability of their findings to U.S. clinical practice [18, 19]. To complement the current evidence on the effectiveness of combination therapies based on broader, contemporary US populations, we conducted target trial emulations to evaluate the cardiovascular effectiveness of empagliflozin and GLP-1RA combination therapy in adults with type 2 diabetes.

## Methods

We emulated two target trials separately within three U.S. healthcare databases: Medicare fee-for-service (2013–2020) and two commercial claims databases—Optum’s de-identified Clinformatics^®^ Data Mart Database and Merative™ MarketScan^®^ Commercial and Medicare Databases (2013–2022). We specify details of the target trial emulations in Appendix Table 1. Medicare captures longitudinal data on eligible adults aged ≥ 65 years, while commercial claims contain similar data for commercially insured populations. The Mass General Brigham Institutional Review Board approved the study protocol. Data use agreements were in place.

**Table 1:** Selected baseline characteristics of empagliflozin-GLP-1RA combination therapy relative to alternative combinations: results after applying propensity score matching weights

Characteristics*	Empagliflozin background			GLP-1RA background		
	GLP-1RA *N* = 10,629	DPP-4i *N* = 10,608	SMD	Empagliflozin *N* = 19,727	Sulfonylureas *N* = 19,683	SMD
Demographics						
Age (mean, SD)	61.1 (12.14)	61.1 (12.16)	0.002	59.1 (12.34)	59.1 (12.34)	0.000
Sex, Female	4,638 (43.6%)	4,596 (43.3%)	0.006	9,811 (49.7%)	9,777 (49.7%)	0.001
Race						
White	4,266 (64.7%)	4,263 (64.8%)	0.002	7,920 (68.2%)	7,890 (68.2%)	0.001
Black	733 (11.1%)	730 (11.1%)	0.001	1,361 (11.7%)	1,359 (11.7%)	0.001
Asian	302 (4.6%)	296 (4.5%)	0.004	323 (2.8%)	328 (2.8%)	0.003
Hispanic	864 (13.1%)	861 (13.1%)	0	1,337 (11.5%)	1,332 (11.5%)	0
Other/Unknown	425 (6.4%)	425 (6.5%)	0.001	668 (5.8%)	669 (5.8%)	0.001
Burden of comorbidity/deprivation						
Frailty index	0.15 (0.04)	0.15 (0.04)	0.004	0.15 (0.05)	0.15 (0.05)	0.000
Combined comorbidity score	1.5 (2.15)	1.5 (2.19)	0.000	1.7 (2.22)	1.7 (2.23)	0.001
Social deprivation index**	57.7 (7.28)	57.6 (7.28)	0.011	57.7 (7.18)	57.7 (7.18)	0.000
Cardiovascular comorbidities						
Baseline CVD	3,466 (32.6%)	3,428 (32.3%)	0.006	5,850 (29.7%)	5,755 (29.2%)	0.009
Heart failure	986 (9.3%)	987 (9.3%)	0.001	1,578 (8.0%)	1,572 (8.0%)	0.000
Stroke	778 (7.3%)	782 (7.4%)	0.002	1,229 (6.2%)	1,220 (6.2%)	0.001
Diabetes & other comorbidities						
Diabetic nephropathy	1,233 (11.6%)	1,237 (11.7%)	0.002	2,565 (13.0%)	2,558 (13.0%)	0.000
Diabetic neuropathy	1,932 (18.2%)	1,914 (18.0%)	0.004	3,807 (19.3%)	3,803 (19.3%)	0.001
Diabetic retinopathy	395 (3.7%)	390 (3.7%)	0.002	746 (3.8%)	744 (3.8%)	0.000
CKD stage 3–4	532 (5.0%)	532 (5.0%)	0.000	1,298 (6.6%)	1,282 (6.5%)	0.003
COPD	709 (6.7%)	714 (6.7%)	0.002	1,256 (6.4%)	1,249 (6.3%)	0.001
Morbid obesity	1,513 (14.2%)	1,498 (14.1%)	0.003	4,404 (22.3%)	4,404 (22.4%)	0.001
History of treatment						
Days of background therapy	130.2 (234.62)	124.1(221.67)	0.027	182.4 (278.52)	166.9 (260.84)	0.058
Concomitant medications						
Metformin	8,538 (80.3%)	8,529 (80.4%)	0.002	14,893 (75.5%)	14,869 (75.5%)	0.001
Insulin	2,049 (19.3%)	2,038 (19.2%)	0.002	6,198 (31.4%)	6,178 (31.4%)	0.001
DPP-4i	-	-	-	5,053 (25.6%)	5,020 (25.5%)	0.003
Sulfonylureas	3,897 (36.7%)	3,820 (36.0%)	0.014	-	-	-
Beta-blockers	3,818 (35.9%)	3,791 (35.7%)	0.004	6,728 (34.1%)	6,702 (34.0%)	0.001
CCB	2,773 (26.1%)	2,764 (26.1%)	0.001	5,141 (26.1%)	5,126 (26.0%)	0.000
ACEI/ARB	7,441 (70.0%)	7,413 (69.9%)	0.003	13,586 (68.9%)	13,557 (68.9%)	0.000
Loop diuretics	1,135 (10.7%)	1,138 (10.7%)	0.002	2,362 (12.0%)	2,357 (12.0%)	0.000
Anticoagulants	770 (7.2%)	772 (7.3%)	0.001	1,369 (6.9%)	1,357 (6.9%)	0.002
Statins	7,872 (74.1%)	7,828 (73.8%)	0.006	13,780 (69.9%)	13,761 (69.9%)	0.001
Healthcare resource utilization						
Cardiologist visits	1.1 (2.96)	1.1 (3.02)	0.002	1.1 (3.00)	1.1 (2.84)	0.007
No of creatinine tests	0.1 (0.49)	0.1 (0.51)	0.003	0.1 (0.53)	0.1 (0.50)	0.006
No of HbA1c tests	2.2 (1.93)	2.2 (1.93)	0.006	2.6 (2.28)	2.5 (2.21)	0.019
No of ED visits	0.4 (1.18)	0.4 (1.15)	0.009	0.5 (1.35)	0.5 (1.51)	0.003
No of hospitalization	0.1 (0.39)	0.1 (0.40)	0.001	0.1 (0.44)	0.1 (0.45)	0.004
Laboratory results^†^						
HbA1c (%)	9.1 (1.92)	8.9 (1.83)	0.108	8.8 (1.90)	9.0 (2.26)	0.091
eGFR (mL/min/1.73m^2^)	83.8 (21.52)	83.2 (21.73)	0.028	85.0 (22.13)	83.4 (23.22)	0.068

ACEi: Angiotensin-Converting Enzyme Inhibitor; ARB: Angiotensin II Receptor Blocker; CCB: Calcium Channel Blockers; CKD: Chronic Kidney Diseases; COPD: Chronic Obstructive Pulmonary Disease; CVD: Cardiovascular Disease; DPP-4i: Dipeptidyl-peptidase-4 Inhibitors; eGFR: Estimated Glomerular Filtration Rate; HbA1c: Hemoglobin A1c; SD: Standard deviation; SMD: Standardized Mean Differences

*Categorical variables are presented as number of people (percentage) and continuous variables are presented as mean (standard deviation). **Only available in Medicare

^†^Available in 13% of the population

### Study cohorts

The study population consisted of two cohorts of individuals diagnosed with type 2 diabetes who initiated (i) empagliflozin first, followed by augmentation with either GLP-1RA or dipeptidyl peptidase-4 inhibitors (DPP-4i), and (ii) GLP-1RA first, followed by augmentation with either empagliflozin or second-generation sulfonylureas (SU). We used DPP-4i and SU, commonly used second-line glucose-lowering medications, as active comparators to GLP-1RA and empagliflozin, respectively, to minimize confounding by indication and immortal time bias common in observational studies [20–23]. These treatment sequences emulate clinically relevant decisions regarding selection of one add-on therapy versus another among patients already receiving background treatment, rather than comparisons with no treatment or heterogeneous comparators [18, 19, 24]. This approach helps ensure that both treatment groups have similar indications for intensification and comparable engagement with clinical care [20]. Medications included in the drug classes were listed in Appendix Tables 2 and 3.

**Table 2:** Cardiovascular events after initiating empagliflozin-GLP-1RA combination therapy: results after applying propensity score matching weights

Outcomes	No of events (Rates/1,000 PY)	No of events (Rates/1,000 PY)	HR (95% CI)	RD/1,000PY^§^ (95% CI)
	Empagliflozin + GLP-1RA (*N* = 10,629)	Empagliflozin + DPP-4i (*N* = 10,608)		
Primary outcomes				
Modified MACE*	153 (18.25)	179 (22.85)	0.81 (0.67, 0.97)	-3.7 (-7.1, -0.2)
HHF (specific)^†^ or mortality	90 (10.69)	140 (17.84)	0.61 (0.48, 0.77)	-6.6 (-9.3, -3.9)
Secondary outcomes				
HHF (specific)^†^	30 (3.57)	63 (7.95)	0.46 (0.32, 0.66)	-4.3 (-6.3, -2.5)
HHF (broad)^‡^	219 (26.27)	242 (31.08)	0.87 (0.74, 1.02)	-4.5 (-8.5, -0.5)
MI-Stroke	98 (11.66)	97 (12.40)	0.95 (0.74, 1.22)	-0.4 (-3.2, 2.4)
MI	59 (7.08)	54 (6.85)	1.03 (0.74, 1.43)	0.3 (-1.8, 2.5)
Stroke	38 (4.57)	43 (5.51)	0.85 (0.58, 1.25)	-0.7 (-2.4, 1.1)
All-cause mortality	61 (7.25)	90 (11.46)	0.64 (0.48, 0.85)	-3.4 (-5.3, -1.4)

Outcomes	GLP-1RA + Empagliflozin (*N* = 19,727)	GLP-1RA + sulfonylureas (*N* = 19,683)		
Primary outcomes				
Modified MACE*	178 (14.10)	212 (20.90)	0.68 (0.56, 0.81)	-5.3 (-7.9, -2.6)
HHF (specific) ^†^ or mortality	141 (11.15)	178 (17.48)	0.62 (0.51, 0.76)	-3.7 (-6.1, -1.8)
Secondary outcomes				
HHF (specific) ^†^	53 (4.23)	89 (8.69)	0.50 (0.37, 0.67)	-3.1 (-4.8, -1.5)
HHF (broad)^‡^	334 (26.68)	351 (34.78)	0.79 (0.69, 0.91)	-7.5 (-10.9, -4.1)
MI-Stroke	95 (7.55)	126 (12.43)	0.63 (0.50, 0.80)	-4.4 (-6.7, -2.3)
MI	53 (4.17)	66 (6.51)	0.66 (0.48, 0.90)	-1.9 (-3.3, -0.4)
Stroke	46 (3.60)	64 (6.27)	0.60 (0.42, 0.85)	-2.6 (-4.3, -1.0)
All-cause mortality	94 (7.41)	95 (9.26)	0.77 (0.59, 0.99)	-0.6 (-2.0, 0.7)

CI: confidence intervals; DPP-4i, Dipeptidyl peptidase-4 inhibitors; GLP-1RA, Glucagon-like peptide-1 receptor agonists; HHF, Hospitalization for heart failure; HR: hazard ratio; IR: Incidence rate; ITT: intent-to-treat; MACE, Major adverse cardiovascular events; MI, Myocardial infarction; PS: propensity score; PY: person-years; RD: rate difference

*Modified MACE is defined as a composite of myocardial infarction, ischemic or hemorrhagic stroke, or all-cause mortality

^†^Hospitalization for heart failure (specific) is defined as heart failure diagnosis codes in the primary hospital discharge position

^‡^Hospitalization for heart failure (broad) is defined as heart failure diagnosis codes in all hospital discharge positions

^§^RDs were estimated by pooling across 3 databases using fixed-effects meta-analysis to account for between-database heterogeneity

**Table 3:** Sensitivity analyses for assessing cardiovascular events after initiating empagliflozin-GLP-1RA combination therapy

	Empagliflozin & GLP-1RA combo therapy Number of events (IR/1,000 PY)		Alternative combo therapy of either agent alone Number of events (IR/1,000 PY)		HR (95% CI)	RD/1,000PY§ (95% CI)
Empagliflozin background						
Modified MACE*						
One-year ITT	10,629	141 (16.27)	10,608	178 (20.67)	0.79 (0.65, 0.96)	-4.3 (-7.4, -1.3)
Lag follow-up (earliest background drug refill after augmentation)	10,583	149 (18.66)	10,563	174 (23.24)	0.82 (0.67, 0.99)	-3.7 (-7.3, -0.1)
Lag follow-up (60 days)	9,774	121 (18.29)	9,749	144 (23.38)	0.80 (0.64, 0.98)	-4.1 (-8.1, -0.2)
HHF (specific)^†^ or mortality						
One-year ITT	10,629	95 (11.00)	10,608	140 (16.23)	0.68 (0.54, 0.86)	-5.7 (-8.1, -3.3)
Lag follow-up (earliest background drug refill after augmentation)	10,583	89 (10.59)	10,563	135 (17.20)	0.63 (0.50, 0.79)	-6.2 (-8.8, -3.6)
Lag follow-up (60 days)	9,774	77 (9.35)	9,749	116 (14.85)	0.64 (0.49, 0.82)	-5.2 (-7.6, -2.8)
GLP-1RA background						
Modified MACE*						
One-year ITT	19,727	211 (13.27)	19,683	293 (18.37)	0.72 (0.61, 0.84)	-3.1 (-5.0, -1.1)
Lag follow-up (earliest background drug refill after augmentation)	13,001	138 (13.33)	12,973	179 (20.63)	0.65 (0.53, 0.79)	-5.2 (-8.1, -2.4)
Lag follow-up (60 days)	11,880	110 (10.86)	11,852	144 (16.78)	0.62 (0.50, 0.78)	-5.7 (-9.0, -2.5)
HHF (specific)^†^ or mortality						
One-year ITT	19,727	186 (11.69)	19,683	256 (16.05)	0.72 (0.61, 0.85)	-1.9 (-3.4, -0.3)
Lag follow-up (earliest background drug refill after augmentation)	13,001	105 (10.13)	12,973	145 (16.63)	0.60 (0.47, 0.75)	-3.8 (-6.0, -1.6)
Lag follow-up (60 days)	11,880	91 (8.95)	11,852	122 (14.18)	0.60 (0.47, 0.76)	-3.4 (-5.5, -1.4)

CI: confidence intervals; DPP-4i, Dipeptidyl peptidase-4 inhibitors; GLP-1RA, Glucagon-like peptide-1 receptor agonists; HHF, Hospitalization for heart failure; HR: hazard ratio; IR: Incidence rate; ITT: intent-to-treat; MACE, Major adverse cardiovascular events; MI, Myocardial infarction; PS: propensity score; PY: person-years; RD: rate difference

*Modified MACE is defined as a composite of myocardial infarction, ischemic or hemorrhagic stroke, or all-cause mortality

^†^Hospitalization for heart failure (specific) is defined as heart failure diagnosis codes in the primary hospital discharge position

§ RDs were estimated by pooling across 3 databases using fixed-effects meta-analysis to account for between-database heterogeneity

To ensure we captured patients initiating treatment, we identified those who newly started the study medications after a 6-month baseline period without any dispensing of SGLT2i, GLP-1RA, or DPP-4i (for the empagliflozin cohort) or GLP-1RA, SGLT2i, or SU (for the GLP-1RA cohort). We excluded individuals with type 1, gestational, or secondary diabetes; end-stage kidney disease; acute or chronic pancreatitis; cirrhosis; acute hepatitis; multiple endocrine neoplasia type 2; solid organ transplant; bariatric surgery; or prior dispensing of GLP-1RAs indicated solely for obesity (Saxenda^®^, Wegovy^®^, or Zepbound^®^) during the baseline period up to the augmentation date.

Follow-up started one day after the date of treatment augmentation and continued until the earliest occurrence of the following: a study outcome, end of the study period, end of health plan enrollment, discontinuation of either background or added medications, switching to the comparator drug class within the cohort, undergoing bariatric surgery, or death. To determine continued medication use, we allowed a 60-day grace period after the end of the medication’s days’ supply.

### Outcomes

Primary outcomes included (i) modified 3-point major adverse cardiovascular events (MACE), a composite of MI, ischemic or hemorrhagic stroke, or all-cause mortality, and (ii) a composite of hospitalization for heart failure (HHF, primary hospital discharge diagnosis), or all-cause mortality. We defined these outcomes using ICD-9-CM or ICD-10-CM diagnosis codes in inpatient settings that have been validated and have high specificity or positive predictive values [25–27]. Secondary outcomes included HHF defined more broadly (all hospital discharge positions) and the individual components of the MACE outcome.

### Covariates

Within each database, we captured over 130 baseline covariates. These included age, sex, race/ethnicity, comorbidities (including the combined comorbidity score [28]), frailty index [29, 30], treatment history (duration of background therapy before augmentation), concomitant medications, and healthcare resource utilization, including preventive and screening services. We further quantified neighborhood-level socioeconomic status (SES) using the ZIP code-level social deprivation index in Medicare [31, 32]. In a subset of the population that had laboratory values available (~ 13%), we additionally measured hemoglobin A1c (HbA1c), lipid profile, and estimated glomerular filtration rate (eGFR).

### Statistical analyses

To improve comparability across treatment groups, we conducted propensity score (PS) matching weights (MW) within each cohort using the covariates described above [33, 34]. For each comparison, and separately within each database, we predicted the PS — the probability of augmenting treatment with GLP-1RA relative to DPP-4i (in the empagliflozin cohort), or with empagliflozin relative to SU (in the GLP-1RA cohort)—using multivariable logistic regression. We then assigned weights based on the PS: the weight for each individual was calculated as the smaller of the following numbers (PS or 1-PS), divided by the PS in the treated group and 1-PS in the reference group. This weighting method helped improve covariate balance between treatment groups, allowing for estimation of treatment effects in the population with shared baseline characteristics [34, 35]. 

We assessed covariate balance before and after MW by standardized mean differences (SMD), with values less than 0.1 considered acceptable for confounding adjustment [36]. We estimated hazard ratios (HRs) using stratified Cox proportional hazards models, with stratification by database to account for between-database differences. Rate differences (RDs) per 1,000 person-years were calculated as the difference in PS-weighted event rates between treatment groups within each database. Confidence intervals for RDs were obtained via stratified bootstrap resampling (1,000 iterations), sampling performed with replacement within each database [37]. Database-specific RD estimates were then pooled using fixed-effects meta-analysis with inverse variance weighting [37]. We estimated the cumulative risk of the outcome over time using Kaplan-Meier methods and plotted the results using Cumulative Incidence Function (CIF) curves [37]. 

### Sensitivity analyses

To reduce potential misclassification of combination therapy exposure, we conducted two sensitivity analyses. First, we initiated follow-up 60 days after the date of augmentation to allow time for patients to fill prescriptions for both empagliflozin and a GLP-1RA and to exclude those who switched rather than augmented therapy. Second, we initiated follow-up at the earliest dispensing date of the background therapy after augmentation, ensuring that exposure classification required observed dispensing of both medications. To account for informative censoring and allow longer follow-up, we conducted intention-to-treat (ITT) analyses that did not censor for treatment changes up to one year of follow-up. To assess the impact of potential unmeasured confounding due to SES and frailty on our estimates, we conducted a quantitative bias analysis [37–40]. Specifically, we modeled the impact of strong associations between each confounder and cardiovascular outcomes (relative risks: 1.0-3.25)—assuming that patients receiving empagliflozin–GLP-1RA combination therapy had higher SES and lower frailty compared with those receiving combinations involving either therapy alone [39, 40]. We then calculated bias-adjusted relative risks and compared them to our observed estimates to evaluate the robustness of our findings [37, 38].

### Subgroup analyses

We conducted separate analyses within (i) age (< 65 years and ≥ 65 years), (ii) sex (male and female patients), and (iii) individuals with and without baseline CVD. We defined baseline CVD as the presence of diagnostic codes for any of the following conditions: heart failure, MI, angina, coronary atherosclerosis or ischemic heart disease, coronary revascularization, stroke, peripheral arterial disease or surgery, or lower extremity amputations.

## Results

### Study cohorts and patient characteristics

In the empagliflozin cohort, we identified 26,775 empagliflozin initiators who added GLP-1RA vs. 12,884 empagliflozin initiators who added DPP-4i. In the GLP-1RA cohort, we identified 31,073 GLP-1RA initiators who added empagliflozin vs. 26,558 GLP-1RA initiators who added SU (Appendix Figs. 3 and 4 show consort diagrams for the two cohorts by each of the three databases included in the study).

Before applying MW, empagliflozin initiators who added GLP-1RA, compared with those who added DPP-4i, were younger (mean age: 58 vs. 62 years) and more likely to have recorded diagnosis codes for severe obesity (23.5% vs. 12.1%). They were also more likely to have a longer duration of background therapy before augmentation (mean: 183 vs. 110 days) and to be treated with insulin at baseline (35.3% vs. 16.3%). The prevalence of baseline CVD was similar between treatments (32.5% vs. 32.9%) (Appendix Table 4).

**Table 4:** Subgroup analyses for the cardiovascular effectiveness of combined empagliflozin + GLP-1RA vs. alternative combination therapy*

Empagliflozin background	*N*	Events (IR/1,000 PY)	*N*	Events (IR/1,000 PY)	HR (95% CI)	RD (95% CI)
	Empagliflozin + GLP-1RA		Empagliflozin + DPP-4i			
Modified MACE						
Age 65 + years	4910	112 (31.3)	4897	130 (37.5)	0.83 (0.66, 1.04)	-6.0 (-13.6, 1.7)
Age under 65 years	5620	39 (8.3)	5605	47 (10.9)	0.74 (0.51, 1.07)	-2.3 (-5.8, 1.3)
Male	5859	83 (17.2)	5861	91 (20.3)	0.83 (0.64, 1.07)	-1.9 (-6.3, 2.5)
Female	4610	68 (20.0)	4590	81 (25.3)	0.82 (0.61, 1.09)	-6.4 (-12.1, -0.7)
Baseline CVD	3393	91 (35.5)	3387	111 (45.2)	0.78 (0.61, 0.99)	-6.5 (-15.8, 2.8)
No baseline CVD	7088	60 (10.5)	7068	69 (13.0)	0.81 (0.59, 1.11)	-2.6 (-5.9, 0.7)
HHF (specific) or mortality						
Age 65 + years	4910	77 (21.4)	4897	109 (31.35)	0.69 (0.53, 0.89)	-10.5 (-17.1, -3.9)
Age under 65 years	5620	13 (2.7)	5605	28 (6.56)	0.36 (0.22, 0.60)	-3.7 (-6.0, -1.3)
Male	5859	50 (10.3)	5861	74 (16.44)	0.62 (0.45, 0.84)	-5.1 (-8.4, -1.8)
Female	4610	38 (11.0)	4590	63 (19.51)	0.56 (0.39, 0.80)	-8.3 (-12.8, -3.9)
Baseline CVD	3393	58 (22.2)	3387	97 (39.58)	0.55 (0.42, 0.73)	-14.9 (-22.4, -7.5)
No baseline CVD	7088	30 (5.3)	7068	42 (8.01)	0.61 (0.41, 0.93)	-3.4 (-5.9, -0.9)

GLP-1RA background	GLP-1RA + Empagliflozin		GLP-1RA + Sulfonylureas			
Modified MACE						
Age 65 + years	7762	125 (26.3)	7743	148 (37.8)	0.69 (0.56, 0.85)	-10.7 (-17.1, -4.2)
Age under 65 years	11,705	50 (6.5)	11,703	60 (9.9)	0.66 (0.47, 0.92)	-3.4 (-6.1, -0.6)
Male	9756	95 (14.1)	9737	117 (22.0)	0.65 (0.51, 0.82)	-6.1 (-9.9, -2.4)
Female	9734	81 (14.3)	9716	94 (20.0)	0.71 (0.54, 0.93)	-4.6 (-8.2, -0.9)
Baseline CVD	5682	103 (28.8)	5659	135 (47.3)	0.62 (0.49, 0.78)	-18.4 (-26.7, -10.1)
No baseline CVD	13,817	75 (8.5)	13,823	75 (10.3)	0.82 (0.61, 1.09)	-1.7 (-4.0, 0.7)
HHF (specific) or mortality						
Age 65 + years	7762	107 (22.4)	7743	140 (35.6)	0.62 (0.49, 0.77)	-11.1 (-17.3, -5.0)
Age under 65 years	11,705	33 (4.27)	11,703	38 (6.1)	0.66 (0.43, 1.02)	-2.3 (-4.3, -0.2)
Male	9756	76 (11.2)	9737	94 (17.7)	0.63 (0.48, 0.82)	-2.2 (-5.1, 0.6)
Female	9734	63 (11.0)	9716	84 (17.8)	0.60 (0.45, 0.81)	-5.5 (-8.8, -2.1)
Baseline CVD	5682	89 (24.8)	5659	137 (48.1)	0.52 (0.41, 0.66)	-20.1 (-27.6, -12.5)
No baseline CVD	13,817	50 (5. 7)	13,823	39 (5.4)	1.00 (0.68, 1.47)	0.0 (-1.7, 1.7)

CI, confidence interval; CVD, Cardiovascular disease; DPP-4i, Dipeptidyl peptidase-4 inhibitors; GLP-1RA, Glucagon-like peptide-1 receptor agonists; HHF, Hospitalization for heart failure; HR: hazard ratio; IR: Incidence rate; ITT: intent-to-treat; MACE, Major adverse cardiovascular events; N, number of individuals; PY: person-years; RD: rate difference

*Subgroup analyses were exploratory. Multiple comparisons were not adjusted for; therefore, some findings may be due to chance and should be interpreted cautiously

In the GLP-1RA cohort, GLP-1RA initiators who added empagliflozin, compared with those adding SU, were younger (mean age: 58 vs. 61 years), had similar distributions of baseline CVD (32.0% vs. 31.1%), and were less likely to have chronic kidney diseases (5.4% vs. 9.6% stages 3–4). They were more likely to have diagnoses of severe obesity (25.8% vs. 20.2%), a longer duration of background therapy before augmentation (215 vs. 154 days), and to have received insulin at baseline (42.8% vs. 27.3%) (Appendix Table 5). After MW, all these differences were minimal with small SMDs, including laboratory parameters available in a subset of the population and not included in the PS estimation (Table 1).

In the empagliflozin cohort, median treatment durations after augmentation were similar between those adding GLP-1RA vs. DPP-4i (median 4 months; interquartile range, IQR: 3, 12 months). The average was approximately 9 months. The most common reasons for censoring were discontinuation of background (26–31%) and augmented therapies (18–28%) (Appendix Table 6).

In the GLP-1RA cohort, median treatment durations after augmentation were similar between those adding empagliflozin and those adding SU (median: 4 months; IQR: 2, 10 months). However, the average follow-up was slightly longer for the empagliflozin group (8 vs. 6 months). The most common censoring reason was discontinuation of background GLP-1RA (39–44% across cohorts), followed by discontinuation of augmented therapies (21%).

### Absolute and relative risks of primary and secondary outcomes

In the empagliflozin cohort, after applying MW, we observed 18 modified MACE events per 1,000 person-years among initiators who added a GLP-1RA, compared with 23 events per 1,000 person-years among those who added a DPP-4i. The weighted HR (95% CI) was 0.81 (0.67, 0.97), and RD per 1,000 person-years (95% CI) was − 3.7 (-7.1, -0.2). The augmentation with GLP-1RA was also associated with lower HHF-mortality (11 vs. 18 per 1,000 person-years): HR (95% CI) of 0.61 (0.48, 0.77) and RD per 1,000 person-years (95% CI) of -6.6 (-9.3, -3.9). The rates of MI and stroke events were similar between the two combination therapies, while the rates of HHF and all-cause mortality were lower with the empagliflozin-GLP-1RA combination therapy. The estimate for HHF events defined more broadly was closer to the null (Table 2).

In the GLP-1RA cohort, after applying MW, we observed 14 modified MACE events per 1,000 person-years among initiators who added empagliflozin, compared with 21 events among those who added SU [HR (95% CI) of 0.68 (0.56, 0.81) and RD (95% CI) of -5.3 (-7.9, -2.6)]. Augmentation with empagliflozin was also associated with lower rates of HHF-mortality (11 vs. 17 per 1,000 person-years): [HR (95% CI): 0.62 (0.51, 0.76); RD (95% CI): -3.7 (-6.1, -1.8)]. The estimates were consistent for secondary outcomes, with combined GLP-1RA-empagliflozin initiators having lower rates of HHF (both specific and broad definitions), MI, stroke, and all-cause mortality (Table 2).

**Fig. 1:**
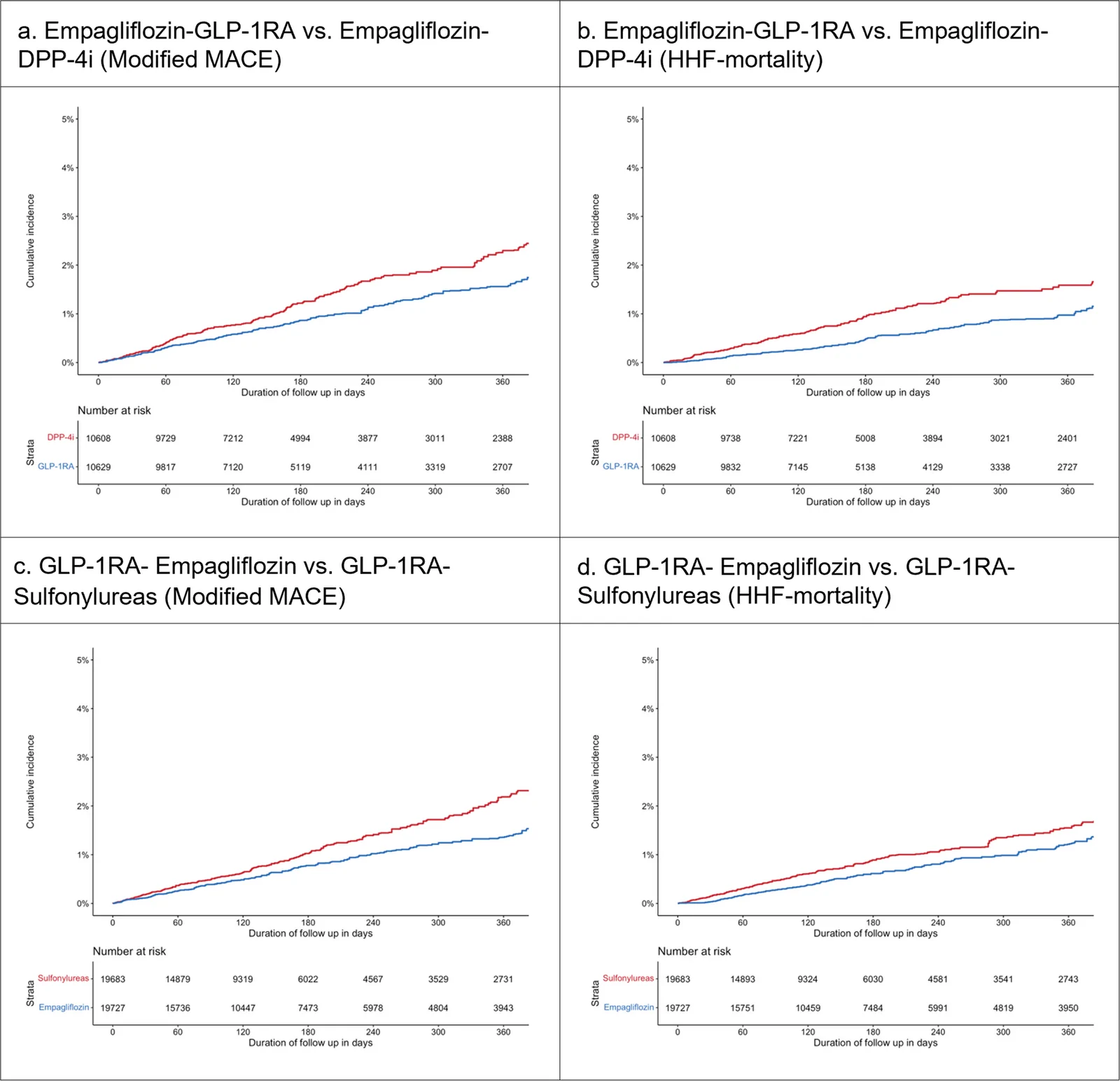
Cumulative risk of cardiovascular outcomes among combination therapy initiators after applying propensity score matching weights. Abbreviations: DPP-4i, Dipeptidyl peptidase-4 inhibitors; GLP-1RA, Glucagon-like peptide-1 receptor agonists; HHF, Hospitalization for heart failure; MACE, Major adverse cardiovascular events. *Modified MACE is defined as a composite of hospitalization for myocardial infarction, ischemic or hemorrhagic stroke, and all-cause mortality. **Log-rank p-values were < 0.05 for all comparisons (panels **a**–**d**)

CIF plots showed lower cumulative risks for modified MACE and HHF-mortality events with empagliflozin and GLP-1RA combination therapies during the first year of follow-up (Fig. 1).

### Sensitivity and subgroup analyses

Analyses varying the start of the follow-up and one-year ITT analyses provided similar risk reductions as in the primary analyses (Table 3). Bias analyses showed that adjusted estimates remained robust even under extreme assumptions about frailty and SES effects and substantial residual imbalances—specifically, higher SES and lower frailty among empagliflozin–GLP-1RA initiators compared with the comparator group (Appendix Fig. 5).

In general, we observed consistent risk reductions across subgroups defined by age, sex, and baseline CVD, although subgroups representing individuals younger than 65 years and no baseline CVD had small numbers of events, which reduced the precision of the estimates. Subgroups that included older, female, and baseline CVD generally had greater magnitudes of absolute risk reductions compared with their younger, male, and no baseline CVD counterparts (Table 4).

## Discussion

In real-world clinical practice among individuals with type 2 diabetes in the US, empagliflozin-GLP-1RA combination therapy was associated with lower rates of cardiovascular events (modified MACE and HHF-mortality) when compared with alternative regimens involving either medication alone. The associations were consistent regardless of the order of treatment initiation and across sensitivity and subgroup analyses. Across cohorts, associations were strongest and most consistent for HHF, whereas patterns for MI, stroke, and all-cause mortality varied by treatment-initiation cohort and comparator regimen. Greater absolute risk reductions were observed among older adults and those with baseline cardiovascular disease—who comprised the majority of the study population—suggesting these groups may derive the greatest benefit from combined therapy.

Our findings are broadly consistent with prior meta-analyses of cardiovascular outcome trials , although several important differences should be noted. First, previous meta-analytic estimates are based on relatively small numbers of patients receiving combination therapy, resulting in limited precision [10–15]. Second, those analyses compared patients receiving both SGLT2i and GLP-1RA with those receiving either therapy alone, whereas our study used DPP-4i or sulfonylurea as active comparators to reduce confounding by indication and better reflect real-world treatment choices. Third, trial participants were not required to remain on both therapies throughout follow-up, whereas we specifically emulated sustained combination treatment after initiation. Fourth, cardiovascular outcome trials preferentially enrolled patients at high cardiovascular risk, while our study reflects broader populations treated in routine healthcare settings.

A previous study using similar U.S. healthcare data reported comparable results; however, the findings were primarily driven by canagliflozin (62% of SGLT2i use) and focused only on GLP-1RA initiators (60% on liraglutide) who added either SGLT2i or SU [17]. Another study from the UK reported that combined use of SGLT2i and GLP-1RA was associated with a 30% reduction in the risk of MACE and cerebrovascular events compared with use of neither medication [18]. However, residual bias is possible since patients receiving combination therapy may have had more advanced disease or higher baseline risk than those receiving neither medication [18, 24]. Another study using the UK primary care data reported similar cardiovascular risk reductions in line with our findings [19]. However, the use of chronic medication users might introduce selection bias since prevalent drug users are often long-term survivors of earlier adverse outcomes [37, 41]. By contrast, our study emulates clinical decision-making by comparing patients initiating relevant treatment combinations to ensure that both treatment groups were equally exposed to clinical care and were deemed to warrant additional medication. Additionally, findings from the real-world data in the UK may not translate to the US due to differences in formulary restrictions, prescribing practices, and provider preferences [19]. 

Notably, the observed mortality benefit in our study was larger than that reported in trial-based meta-analyses of either drug class alone [42, 43]. This may reflect differences in the underlying population—ours being older and generally sicker—as well as differences in treatment exposure, since our analysis estimated the effect of concurrent combination therapy rather than monotherapy versus placebo. Moreover, whereas most trials focus on cardiovascular mortality, our findings pertain to all-cause mortality, potentially capturing broader benefits beyond heart failure stemming from the pleiotropic effects of GLP-1RAs and SGLT2is in high-risk individuals [44]. 

Our study has several implications. Firstly, while the American Diabetes Association Standards of Care have increasingly emphasized the early use of combined SGLT2i and GLP-1RA to optimize cardiovascular outcomes, the evidence on their additive cardiovascular effectiveness is limited [1, 2, 45]. Trials evaluating the clinical benefits of combined SGLT2i and GLP-1RA therapies are unlikely to be conducted in the near future due to cost and feasibility concerns. Our study fills this gap in evidence by complementing existing guidelines.

Secondly, despite their potential benefits, combination therapies entail added medication burden, higher risks of adverse events, and increased cost. Understanding the extent to which these regimens improve cardiovascular outcomes is therefore critical to balancing benefits and risks and to optimizing efficient, personalized care for patients with complex health conditions [46–49]. 

Thirdly, our study includes broad patient populations in US routine care settings, allowing us to address questions in clinically relevant populations that are often excluded from cardiovascular outcome trials, including older adults, women, racial/ethnic minorities, and those with lower SES [50]. 

### Limitations

Our study has limitations. First, residual confounding is possible given the lack of randomization. To mitigate this, we restricted comparisons to patients initiating empagliflozin-GLP-1RA combination therapy versus alternative combination regimens and performed extensive adjustment for over 130 covariates, including proxies for socioeconomic status, frailty, diabetes severity, and renal disease, along with bias analyses assessing the potential impact of unmeasured confounding [51]. Although some clinically relevant laboratory measures were available only in a subset of patients and therefore were not included in the primary propensity score models, post hoc balance assessments were generally reassuring.

Second, follow-up was relatively short, reflecting real-world patterns in which patients remained on combination therapy for less than one year before discontinuation or switching. However, cumulative incidence patterns for MACE and HHF-mortality were consistent with prior clinical trial evidence, and results were similar when we extended the follow-up to one year in ITT analyses [6, 7]. 

Third, exposure misclassification is possible, particularly because medication stockpiling or switching may be misclassified as concurrent use. To address this, we varied the start of follow-up to require evidence of dispensing for both empagliflozin and GLP-1RA, thereby applying a more restrictive definition of sustained concomitant use. Although this reduced the number of eligible patients, particularly in the GLP-1RA-background cohort, the resulting estimates remained highly consistent with the primary analyses in direction, magnitude, and precision, supporting the robustness of the main findings. Our study focused on empagliflozin in combination with GLP-1RA as a class effect, and the evaluation of combination with individual GLP-1RA agents should be considered in future studies.

Fourth, outcome misclassification is possible in claims-based data. Therefore, we prioritized outcome definitions with higher specificity, which are expected to yield less biased relative measures of association [37]. For example, estimates for hospitalization for heart failure using broader definitions were numerically closer to the null than those using more specific definitions, potentially reflecting greater sensitivity but increased non-differential misclassification. These findings should be interpreted in the context of the trade-off between specificity and sensitivity in administrative data. Associations for individual components such as MI, stroke, and mortality varied across cohorts. These differences may reflect heterogeneity in treatment mechanisms, comparator selection, lower event rates with reduced statistical precision, and greater susceptibility to outcome misclassification in claims data. Accordingly, component-specific findings should be interpreted with caution.

## Conclusion

In this study emulating two target trials among individuals with type 2 diabetes in US routine care, augmentation with GLP-1RA among empagliflozin initiators, and augmentation with empagliflozin among GLP-1RA initiators, were each associated with a lower risk of modified MACE and the composite of HHF or all-cause mortality within one year compared with alternative add-on therapies (DPP-4i and SU, respectively). Findings were consistent across patient subgroups and sensitivity analyses. These results help address an important evidence gap regarding the cardiovascular effects of combination therapies, particularly given the limited feasibility of dedicated RCTs. Although supportive rather than definitive, our findings suggest that use of combined glucose-lowering agents with established cardioprotective benefits based on individual cardiovascular risk may be an important consideration in the management of type 2 diabetes in routine clinical practice. Decisions regarding empagliflozin–GLP-1RA combination therapy should be individualized and considered alongside other evidence-based cardiorenal risk-reduction strategies.

## Supplementary Information

Below is the link to the electronic supplementary material.

Supplementary Material 1

## Data Availability

A data use agreement is required for each of the data sources in this study. These data use agreements do not permit the authors to share patient level source data or data derivatives with individuals and institutions not covered under the data use agreements. The databases used in this study are accessible to other researchers by contacting the data providers and acquiring data use agreements or licenses.

## Abbreviations

ASCVD: Atherosclerotic cardiovascular disease
CIF: Cumulative Incidence Function
CVD: Cardiovascular disease
DPP-4i: Dipeptidyl peptidase-4 inhibitors
eGFR: Estimated glomerular filtration rate
GLP-1RA: Glucagon-like peptide-1 receptor agonists
HbA1c: Hemoglobin A1c
HF: Heart failure
HHF: Hospitalization for heart failure
HR: Hazard ratio
ICD-9-CM/ICD-10-CM: International Classification of Diseases, Clinical Modification
IQR: Interquartile range
ITT: Intention-to-treat
MACE: Major adverse cardiovascular events
MI: Myocardial infarction
MW: Matching weights
PS: Propensity score
RCTs: Randomized clinical trials
RD: Rate difference
SES: Socioeconomic status
SGLT2i: Sodium-glucose cotransporter 2 inhibitors
SMD: Standardized mean differences
SU: Sulfonylureas
