# Supplementary material for: Cardiovascular outcomes of empagliflozin-GLP-1RA combination therapy in type 2 diabetes: EMPRISE study
Source: Cardiovasc Diabetol. 2026 Jul 12;25:300. doi: 10.1186/s12933-026-03287-w (PMC13644152; doi:10.1186/s12933-026-03287-w)
Supplement: Supplementary file 1 [file 12933_2026_3287_MOESM1_ESM.docx]

**Supplementary Materials**

**Title:** Cardiovascular Outcomes of Empagliflozin-GLP-1RA Combination Therapy in Type 2 Diabetes: EMPRISE study

**Short Title:** Cardiovascular benefits of empagliflozin-GLP-1RA

Phyo T. Htoo, MD, PhD^a^, Hanseul Cho, MD, MPH^a^, Julie M. Paik, MD, ScD^a^, Katsiaryna Bykov, PharmD, ScD^a^, Brendan M. Everett, MD^b^, Robert J. Glynn, ScD, PhD^a^, Christina Shay, PhD^c^, Niklas Schmedt, DrPH^c^, Deborah J. Wexler, MD, MSc^d^, Elisabetta Patorno, MD, DrPH^a^

**Table of Contents**

[***Appendix Table 1. Target trial emulation	2***](#_Toc199509651)

[***Appendix Table 2. Distribution of glucose-lowering agents involved in the combination therapy among empagliflozin background therapy initiators	4***](#_Toc199509652)

[***Appendix Table 3. Distribution of glucose-lowering agents involved in the combination therapy among GLP-1RA background therapy initiators	5***](#_Toc199509653)

[***Appendix Table 4. Baseline characteristics of empagliflozin initiators who added GLP-1RA or DPP-4i	6***](#_Toc199509654)

[***Appendix Table 5. Baseline characteristics of GLP-1RA initiators who added empagliflozin or sulfonylureas	12***](#_Toc199509655)

[***Appendix Table 6. Follow-up time and censoring reasons after applying matching weights	19***](#_Toc199509656)

[***Appendix Figure 1. Study design scheme for combination therapies initiating empagliflozin first	21***](#_Toc199509657)

[***Appendix Figure 2. Study design scheme for combination therapies initiating GLP-1RA first	22***](#_Toc199509658)

[***Appendix Figure 3. Flow chart Empa background	23***](#_Toc199509659)

[***Appendix Figure 4. Flow chart: GLP-1RA background	25***](#_Toc199509660)

[***Appendix Figure 5. Observed and bias-adjusted relative risks demonstrating the impact of strong unmeasured confounding by frailty and SES	27***](#_Toc199509661)

**Appendix Table 1. Target trial emulation**

| **Component** | **Target trials** | **Real-world trial emulations** |
| --- | --- | --- |
| Eligibility | We emulated two target trials. In the 1^st^ trial, individuals were treatment naïve to SGLT2i, GLP-1RA, and DPP-4i and **initiated empagliflozin before augmenting with either GLP-1RA or DPP-4i**. The date of augmentation is the randomization date.  In the 2^nd^ trial, individuals were treatment naïve to SGLT2i, GLP-1RA, and SU and **initiated GLP-1RA before augmenting with either empagliflozin or SU**. The date of augmentation is the randomization date. Eligibility criteria are evaluated during 6 months before background drug initiation.  Inclusion criteria:   - Type 2 diabetes - Age 18+ years (65+ years in Medicare population) - Continuous enrollment in commercial plans (or Medicare parts A, B, and D) for 6 months   Exclusion criteria:   - Type 1, gestational, or secondary diabetes - End-stage kidney disease - History of nursing home admission | Same as target trial |
| Treatment strategies | 1. Initiation of empagliflozin first, followed by augmentation with  (1) GLP-1RA  (2) DPP-4i  2. Initiation of GLP-1RA first, followed by augmentation with  (1) empagliflozin  (2) SU | Same as target trial |
| Treatment assignment | Randomized (1:1) non-blinded | Non-blinded and assumed to be 1:1 randomized within levels of pre-exposure patient characteristics, including demographics, diabetes and cardiovascular-related comorbidities, concomitant medications, frailty index and indicators of frailty, and measures of healthcare utilization across different care settings. |
| Follow-up start (Time 0) | At assignment | Same as target trial |
| Follow-up end | First of occurrence of outcome of interest, death, loss to follow-up, administrative end of follow-up, treatment discontinuation or switch | Same as target trial |
| Outcomes | Two primary outcomes:  (i) Major adverse cardiovascular events (MACE): MI, stroke, or all-cause mortality,  (ii) Hospitalization for heart failure (HHF) or all-cause mortality. | Same as target trial |
| Causal contrast | Per protocol effect (effect of receiving the treatment as stated in the protocol) [primary analysis]  Intention-to-treat effect (effect of being randomized to treatment) [sensitivity analysis] | Observational analogue of per protocol effect (primary analysis) , intention-to-treat- effect (sensitivity analysis) |
| Statistical analysis | Hazard ratios (HR) using Cox proportional hazards model and rate differences (RD) using fixed-effects meta-analysis across Medicare and commercial claims beneficiaries | Same as target trial with adjustment for baseline covariates via propensity score. |

Abbreviations: DPP-4i: Dipeptidyl Peptidase-4 Inhibitor; GLP-1RA: Glucagon-Like Peptide-1 Receptor Agonist; SGLT2i: Sodium–Glucose Cotransporter-2 Inhibitor; SU: Sulfonylurea; T2D: Type 2 Diabetes.

**Appendix Table 2. Distribution of glucose-lowering agents involved in the combination therapy among empagliflozin background therapy initiators**

|  | **Empagliflozin + GLP-1RA** | **Empagliflozin + DPP-4i** |
| --- | --- | --- |
| **Background treatment** |  |  |
| empagliflozin | 9,130 (85.9%) | 10,006 (94.3%) |
| empagliflozin/linagliptin | 322 (3.0%) | 173 (1.6%) |
| empagliflozin/linagliptin/metformin hcl | 14 (0.1%) | NTSR |
| empagliflozin/metformin hcl | 1,162 (10.9%) | 426 (4.0%) |
| **Augmented treatment** |  |  |
| albiglutide | 137 (1.3%) | - |
| dulaglutide | 4,748 (44.7%) | - |
| exenatide | 32 (0.3%) | - |
| exenatide microspheres | 898 (8.5%) | - |
| insulin degludec/liraglutide | 31 (0.3%) | - |
| insulin glargine,human recombinant analog/lixisenatide | 123 (1.2%) | - |
| liraglutide | 1,650 (15.5%) | - |
| lixisenatide | NTSR | - |
| semaglutide | 2,985 (28.1%) | - |
| tirzepatide | 24 (0.2%) | - |
| alogliptin benzoate | - | 87 (0.8%) |
| alogliptin benzoate/metformin hcl | - | 30 (0.3%) |
| alogliptin benzoate/pioglitazone hcl | - | 30 (0.3%) |
| linagliptin | - | 2,817 (26.6%) |
| linagliptin/metformin hcl | - | 297 (2.8%) |
| saxagliptin hcl | - | 377 (3.6%) |
| saxagliptin hcl/metformin hcl | - | 121 (1.1%) |
| sitagliptin phosphate | - | 5,117 (48.2%) |
| sitagliptin phosphate/metformin hcl | - | 1,733 (16.3%) |

Abbreviations: DPP-4i: Dipeptidyl Peptidase-4 Inhibitor; GLP-1RA: Glucagon-Like Peptide-1 Receptor Agonist.

**Appendix Table 3. Distribution of glucose-lowering agents involved in the combination therapy among GLP-1RA background therapy initiators**

|  | **GLP-1RA + empagliflozin** | **GLP-1RA +**  **Sulfonylureas** |
| --- | --- | --- |
| **Background treatment** |  |  |
| albiglutide | 285 (1.4%) | 273 (1.4%) |
| dulaglutide | 8,746 (44.3%) | 8,534 (43.4%) |
| exenatide | 83 (0.4%) | 242 (1.2%) |
| exenatide microspheres | 1,731 (8.8%) | 1,781 (9.0%) |
| insulin degludec/liraglutide | 80 (0.4%) | 72 (0.4%) |
| insulin glargine,human recombinant analog/lixisenatide | 372 (1.9%) | 379 (1.9%) |
| liraglutide | 3,804 (19.3%) | 4,212 (21.4%) |
| lixisenatide | NTSR | NTSR |
| semaglutide | 4,625 (23.4%) | 4,189 (21.3%) |
| **Augmented treatment** |  |  |
| empagliflozin | 17,326 (87.8%) | - |
| empagliflozin/linagliptin | 404 (2.0%) | - |
| empagliflozin/linagliptin/metformin hcl | 33 (0.2%) | - |
| empagliflozin/metformin hcl | 1,963 (9.9%) | - |
| glimepiride | - | 8,647 (43.9%) |
| glipizide | - | 9,775 (49.7%) |
| glipizide/metformin hcl | - | 227 (1.2%) |
| glyburide | - | 793 (4.0%) |
| glyburide,micronized | - | 33 (0.2%) |
| glyburide/metformin hcl | - | 182 (0.9%) |
| pioglitazone hcl/glimepiride | - | 26 (0.1%) |

Abbreviations: GLP-1RA: Glucagon-Like Peptide-1 Receptor Agonist.

**Appendix Table 4. Baseline characteristics of empagliflozin initiators who added GLP-1RA or DPP-4i**

|  | **Before weighting** | |  | **After weighting** | |  |
| --- | --- | --- | --- | --- | --- | --- |
| **Characteristic** | **GLP-1RA** | **DPP-4i** | **SMD** | **GLP-1RA** | **DPP-4i** | **SMD** |
|  | **N=26,775** | **N=12,884** |  | **N=10,629** | **N=10,608** |  |
| ***Demographics*** |  |  |  |  |  |  |
| Age, mean(SD) | 58.4(11.86) | 62.4(12.43) | 0.33 | 61.1(12.14) | 61.1(12.16) | 0.002 |
| Sex, F | 11,803 (44.1%) | 5,622 (43.6%) | 0.009 | 4,638 (43.6%) | 4,596 (43.3%) | 0.006 |
| Race |  |  |  |  |  |  |
| White | 10,130 (67.0%) | 5,155 (61.7%) | 0.111 | 4,266 (64.7%) | 4,263 (64.8%) | 0.002 |
| Black | 1,775 (11.7%) | 912 (10.9%) | 0.026 | 733 (11.1%) | 730 (11.1%) | 0.001 |
| Asian | 456 (3.0%) | 588 (7.0%) | 0.184 | 302 (4.6%) | 296 (4.5%) | 0.004 |
| Hispanic | 1,887 (12.5%) | 1,135 (13.6%) | 0.033 | 864 (13.1%) | 861 (13.1%) | 0 |
| Other/Unknown | 873 (5.8%) | 565 (6.8%) | 0.041 | 425 (6.4%) | 425 (6.5%) | 0.001 |
| Year of cohort entry |  |  |  |  |  |  |
| 2014-2017 | 3,502 (13.1%) | 3,462 (26.9%) | 0.35 | 2,340 (22.0%) | 2,327 (21.9%) | 0.002 |
| 2018 | 2,643 (9.9%) | 1,811 (14.1%) | 0.129 | 1,443 (13.6%) | 1,442 (13.6%) | 0.001 |
| 2019 | 5,045 (18.8%) | 2,703 (21.0%) | 0.054 | 2,279 (21.4%) | 2,268 (21.4%) | 0.002 |
| 2020 | 5,858 (21.9%) | 2,574 (20.0%) | 0.047 | 2,306 (21.7%) | 2,303 (21.7%) | 0 |
| 2021 | 5,394 (20.1%) | 1,397 (10.8%) | 0.259 | 1,353 (12.7%) | 1,356 (12.8%) | 0.001 |
| 2022 | 4,333 (16.2%) | 937 (7.3%) | 0.28 | 908 (8.5%) | 913 (8.6%) | 0.002 |
| ***Burden of comorbidity/deprivation*** |  |  |  |  |  |  |
| Combined comorbidity score, mean(SD) | 1.7(2.15) | 1.5(2.23) | 0.055 | 1.5(2.15) | 1.5(2.19) | 0 |
| Frailty score, mean(SD) | 0.15 (0.04) | 0.15 (0.04) | 0.048 | 0.15 (0.04) | 0.15 (0.04) | 0.004 |
| SES index score, mean(SD) | 57.7(7.28) | 57.8(7.28) | 0.011 | 57.7(7.28) | 57.6(7.28) | 0.011 |
| ***Cardiovascular comorbidities*** |  |  |  |  |  |  |
| Baseline CVD | 8,690 (32.5%) | 4,243 (32.9%) | 0.01 | 3,466 (32.6%) | 3,428 (32.3%) | 0.006 |
| Heart Failure (before cohort entry) | 2,675 (10.0%) | 1,210 (9.4%) | 0.02 | 986 (9.3%) | 987 (9.3%) | 0.001 |
| Heart Failure (between cohort entry and date of augmentation) | 1,346 (5.0%) | 477 (3.7%) | 0.065 | 416 (3.9%) | 413 (3.9%) | 0.001 |
| Unstable Angina | 975 (3.6%) | 444 (3.4%) | 0.011 | 379 (3.6%) | 375 (3.5%) | 0.001 |
| MI | 958 (3.6%) | 395 (3.1%) | 0.029 | 334 (3.1%) | 327 (3.1%) | 0.003 |
| Old MI/MI sequalae | 808 (3.0%) | 378 (2.9%) | 0.005 | 309 (2.9%) | 311 (2.9%) | 0.001 |
| Coronary procedures | 992 (3.7%) | 414 (3.2%) | 0.027 | 356 (3.3%) | 350 (3.3%) | 0.003 |
| PAD | 1,164 (4.3%) | 689 (5.3%) | 0.047 | 532 (5.0%) | 534 (5.0%) | 0.001 |
| Stroke (before cohort entry) | 1,782 (6.7%) | 1,023 (7.9%) | 0.049 | 778 (7.3%) | 782 (7.4%) | 0.002 |
| Stroke (between cohort entry and date of augmentation) | 775 (2.9%) | 303 (2.4%) | 0.034 | 262 (2.5%) | 259 (2.4%) | 0.001 |
| Stable angina (before cohort entry) | 5,962 (22.3%) | 2,940 (22.8%) | 0.013 | 2,394 (22.5%) | 2,383 (22.5%) | 0.002 |
| Stable angina (between cohort entry and date of augmentation) | 3,145 (11.7%) | 1,181 (9.2%) | 0.084 | 1,052 (9.9%) | 1,049 (9.9%) | 0.001 |
| History Coronary Procedure | 1,608 (6.0%) | 725 (5.6%) | 0.016 | 603 (5.7%) | 607 (5.7%) | 0.002 |
| Late Effects Of CBVD | 713 (2.7%) | 311 (2.4%) | 0.016 | 257 (2.4%) | 260 (2.5%) | 0.002 |
| ***Diabetes and other comorbidities*** |  |  |  |  |  |  |
| Diabetic Nephropathy | 3,432 (12.8%) | 1,498 (11.6%) | 0.036 | 1,233 (11.6%) | 1,237 (11.7%) | 0.002 |
| Diabetic Neuropathy | 5,420 (20.2%) | 2,279 (17.7%) | 0.065 | 1,932 (18.2%) | 1,914 (18.0%) | 0.004 |
| Diabetic Retinopathy | 1,105 (4.1%) | 476 (3.7%) | 0.022 | 395 (3.7%) | 390 (3.7%) | 0.002 |
| Diabetes Other Complications | 2,380 (8.9%) | 988 (7.7%) | 0.044 | 836 (7.9%) | 832 (7.8%) | 0.001 |
| Diabetes Peripheral Circulation | 2,080 (7.8%) | 941 (7.3%) | 0.018 | 772 (7.3%) | 775 (7.3%) | 0.002 |
| MASLD | 1,318 (4.9%) | 511 (4.0%) | 0.046 | 433 (4.1%) | 440 (4.2%) | 0.004 |
| All Cancer | 1,342 (5.0%) | 802 (6.2%) | 0.053 | 614 (5.8%) | 616 (5.8%) | 0.001 |
| Anxiety | 3,204 (12.0%) | 1,188 (9.2%) | 0.089 | 1,047 (9.9%) | 1,033 (9.7%) | 0.004 |
| Asthma | 1,699 (6.3%) | 666 (5.2%) | 0.051 | 581 (5.5%) | 583 (5.5%) | 0.001 |
| Valve Disorders | 1,506 (5.6%) | 873 (6.8%) | 0.048 | 661 (6.2%) | 665 (6.3%) | 0.002 |
| Other CVD | 2,536 (9.5%) | 1,210 (9.4%) | 0.003 | 981 (9.2%) | 979 (9.2%) | 0 |
| Other Cardiac Dysrhythmia | 2,010 (7.5%) | 960 (7.5%) | 0.002 | 775 (7.3%) | 775 (7.3%) | 0.001 |
| Atrial Fibrillation | 1,645 (6.1%) | 894 (6.9%) | 0.032 | 706 (6.6%) | 708 (6.7%) | 0.001 |
| Cardiac Conduction Disorders | 766 (2.9%) | 368 (2.9%) | 0 | 292 (2.8%) | 294 (2.8%) | 0.001 |
| Cardiomyopathy | 845 (3.2%) | 347 (2.7%) | 0.027 | 284 (2.7%) | 286 (2.7%) | 0.002 |
| AKI | 648 (2.4%) | 324 (2.5%) | 0.006 | 248 (2.3%) | 251 (2.4%) | 0.002 |
| CKD stages 1-2 | 727 (2.7%) | 405 (3.1%) | 0.025 | 313 (2.9%) | 313 (3.0%) | 0.001 |
| CKD stages 3-4 | 1,274 (4.8%) | 668 (5.2%) | 0.02 | 532 (5.0%) | 532 (5.0%) | 0 |
| CKD unspecified | 616 (2.3%) | 328 (2.5%) | 0.016 | 247 (2.3%) | 247 (2.3%) | 0 |
| Continuous Glucose Monitoring | 844 (3.2%) | 204 (1.6%) | 0.103 | 190 (1.8%) | 184 (1.7%) | 0.004 |
| COPD | 1,732 (6.5%) | 927 (7.2%) | 0.029 | 709 (6.7%) | 714 (6.7%) | 0.002 |
| Dementia | 323 (1.2%) | 308 (2.4%) | 0.089 | 190 (1.8%) | 188 (1.8%) | 0.001 |
| Depression | 3,379 (12.6%) | 1,282 (10.0%) | 0.084 | 1,139 (10.7%) | 1,125 (10.6%) | 0.004 |
| Disorders Ca Phos Mg Balance | 747 (2.8%) | 359 (2.8%) | 0 | 296 (2.8%) | 293 (2.8%) | 0.001 |
| Disorders Electrolyte Balance | 1,282 (4.8%) | 661 (5.1%) | 0.016 | 521 (4.9%) | 518 (4.9%) | 0.001 |
| Disorders Of Fluid | 565 (2.1%) | 291 (2.3%) | 0.01 | 221 (2.1%) | 223 (2.1%) | 0.001 |
| Dorsopathies | 5,785 (21.6%) | 2,587 (20.1%) | 0.038 | 2,214 (20.8%) | 2,186 (20.6%) | 0.005 |
| Drug Abuse Dependence | 358 (1.3%) | 153 (1.2%) | 0.013 | 129 (1.2%) | 129 (1.2%) | 0 |
| Edema | 1,609 (6.0%) | 710 (5.5%) | 0.021 | 605 (5.7%) | 599 (5.6%) | 0.002 |
| Hyperglycemia | 15,035 (56.2%) | 6,307 (49.0%) | 0.145 | 5,367 (50.5%) | 5,365 (50.6%) | 0.002 |
| Hyperlipidemia | 19,858 (74.2%) | 9,722 (75.5%) | 0.03 | 7,980 (75.1%) | 7,954 (75.0%) | 0.002 |
| Hypertension | 20,600 (76.9%) | 10,034 (77.9%) | 0.023 | 8,218 (77.3%) | 8,189 (77.2%) | 0.003 |
| Hypoglycemia | 3,316 (12.4%) | 1,318 (10.2%) | 0.068 | 1,133 (10.7%) | 1,123 (10.6%) | 0.002 |
| Hypothyroidism | 4,075 (15.2%) | 1,990 (15.4%) | 0.006 | 1,634 (15.4%) | 1,611 (15.2%) | 0.005 |
| HONK/DKA | 370 (1.4%) | 144 (1.1%) | 0.024 | 121 (1.1%) | 122 (1.1%) | 0.001 |
| Osteoporosis | 718 (2.7%) | 601 (4.7%) | 0.106 | 407 (3.8%) | 404 (3.8%) | 0.001 |
| Liver Disease | 425 (1.6%) | 251 (1.9%) | 0.027 | 172 (1.6%) | 174 (1.6%) | 0.001 |
| Major Bleeding | 1,409 (5.3%) | 686 (5.3%) | 0.003 | 549 (5.2%) | 546 (5.1%) | 0.001 |
| Obstructive Sleep Apnea | 5,009 (18.7%) | 1,545 (12.0%) | 0.187 | 1,434 (13.5%) | 1,428 (13.5%) | 0.001 |
| Osteoarthritis | 3,654 (13.6%) | 1,894 (14.7%) | 0.03 | 1,508 (14.2%) | 1,492 (14.1%) | 0.004 |
| Proteinuria | 1,121 (4.2%) | 448 (3.5%) | 0.037 | 377 (3.5%) | 385 (3.6%) | 0.004 |
| Sleep Disorders | 1,905 (7.1%) | 812 (6.3%) | 0.032 | 683 (6.4%) | 678 (6.4%) | 0.001 |
| ***Behavioral risk factors*** |  |  |  |  |  |  |
| Smoking | 4,392 (16.4%) | 1,888 (14.7%) | 0.048 | 1,600 (15.1%) | 1,599 (15.1%) | 0 |
| Alcohol Abuse Dependence | 202 (0.8%) | 84 (0.7%) | 0.012 | 71 (0.7%) | 71 (0.7%) | 0 |
| Overweight | 1,699 (6.3%) | 1,097 (8.5%) | 0.083 | 852 (8.0%) | 845 (8.0%) | 0.002 |
| Morbid Obesity | 6,301 (23.5%) | 1,558 (12.1%) | 0.302 | 1,513 (14.2%) | 1,498 (14.1%) | 0.003 |
| Normal Under Wt | 387 (1.4%) | 402 (3.1%) | 0.112 | 250 (2.4%) | 246 (2.3%) | 0.002 |
| Obesity | 5,930 (22.1%) | 2,116 (16.4%) | 0.145 | 1,924 (18.1%) | 1,911 (18.0%) | 0.002 |
| Unspecified Obesity | 7,034 (26.3%) | 2,253 (17.5%) | 0.214 | 2,097 (19.7%) | 2,063 (19.5%) | 0.007 |
| ***History of treatment*** |  |  |  |  |  |  |
| Days of background therapy, mean(SD) | 182.5(287.78) | 109.9(209.86) | 0.288 | 130.2(234.62) | 124.1(221.67) | 0.027 |
| ***Concomitant Medications*** |  |  |  |  |  |  |
| No. glucose-lowering medications |  |  |  |  |  |  |
| 0 | 5,167 (19.3%) | 2,386 (18.5%) | 0.02 | 2,018 (19.0%) | 2,015 (19.0%) | 0 |
| 1 | 12,787 (47.8%) | 6,770 (52.5%) | 0.096 | 5,466 (51.4%) | 5,461 (51.5%) | 0.001 |
| 2 | 7,418 (27.7%) | 3,205 (24.9%) | 0.064 | 2,681 (25.2%) | 2,674 (25.2%) | 0 |
| 3 | 1,403 (5.2%) | 523 (4.1%) | 0.056 | 464 (4.4%) | 458 (4.3%) | 0.002 |
| Metformin | 20,950 (78.2%) | 10,397 (80.7%) | 0.061 | 8,538 (80.3%) | 8,529 (80.4%) | 0.002 |
| Insulin | 9,448 (35.3%) | 2,101 (16.3%) | 0.444 | 2,049 (19.3%) | 2,038 (19.2%) | 0.002 |
| Sulfonylureas | 8,888 (33.2%) | 4,713 (36.6%) | 0.071 | 3,897 (36.7%) | 3,820 (36.0%) | 0.014 |
| TZD | 2,506 (9.4%) | 1,129 (8.8%) | 0.021 | 971 (9.1%) | 959 (9.0%) | 0.003 |
| No. CVD medications |  |  |  |  |  |  |
| 0 | 6,501 (24.3%) | 3,145 (24.4%) | 0.003 | 2,612 (24.6%) | 2,626 (24.8%) | 0.004 |
| 1 | 7,515 (28.1%) | 3,729 (28.9%) | 0.019 | 3,039 (28.6%) | 3,037 (28.6%) | 0.001 |
| 2 | 6,959 (26.0%) | 3,276 (25.4%) | 0.013 | 2,719 (25.6%) | 2,706 (25.5%) | 0.002 |
| 3 | 4,201 (15.7%) | 2,020 (15.7%) | 0 | 1,661 (15.6%) | 1,644 (15.5%) | 0.003 |
| 4 | 1,599 (6.0%) | 714 (5.5%) | 0.018 | 598 (5.6%) | 596 (5.6%) | 0 |
| ACEI/ARB | 18,979 (70.9%) | 8,993 (69.8%) | 0.024 | 7,441 (70.0%) | 7,413 (69.9%) | 0.003 |
| Beta Blockers | 9,971 (37.2%) | 4,602 (35.7%) | 0.032 | 3,818 (35.9%) | 3,791 (35.7%) | 0.004 |
| Calcium Channel Blockers | 6,862 (25.6%) | 3,446 (26.7%) | 0.025 | 2,773 (26.1%) | 2,764 (26.1%) | 0.001 |
| Loop Diuretics | 3,441 (12.9%) | 1,342 (10.4%) | 0.076 | 1,135 (10.7%) | 1,138 (10.7%) | 0.002 |
| Thiazide | 8,123 (30.3%) | 3,697 (28.7%) | 0.036 | 3,140 (29.5%) | 3,099 (29.2%) | 0.007 |
| Other Antihypertensive Meds | 1,483 (5.5%) | 649 (5.0%) | 0.022 | 561 (5.3%) | 549 (5.2%) | 0.005 |
| MRA Potassium Sparing Diuretics | 1,373 (5.1%) | 502 (3.9%) | 0.059 | 436 (4.1%) | 429 (4.0%) | 0.003 |
| Antiarrhythmics | 454 (1.7%) | 212 (1.6%) | 0.004 | 173 (1.6%) | 176 (1.7%) | 0.002 |
| Anticonvulsants | 5,531 (20.7%) | 2,165 (16.8%) | 0.099 | 1,884 (17.7%) | 1,861 (17.5%) | 0.005 |
| Antidepressants | 8,273 (30.9%) | 3,086 (24.0%) | 0.156 | 2,739 (25.8%) | 2,711 (25.6%) | 0.005 |
| Antiparkinsonian Meds | 705 (2.6%) | 274 (2.1%) | 0.033 | 235 (2.2%) | 232 (2.2%) | 0.002 |
| Anticoagulants | 2,012 (7.5%) | 940 (7.3%) | 0.008 | 770 (7.2%) | 772 (7.3%) | 0.001 |
| Antiplatelet Agents | 3,282 (12.3%) | 1,524 (11.8%) | 0.013 | 1,250 (11.8%) | 1,237 (11.7%) | 0.003 |
| Antipsychotics | 854 (3.2%) | 361 (2.8%) | 0.023 | 306 (2.9%) | 303 (2.9%) | 0.002 |
| Anxiolytics Hypnotics | 2,185 (8.2%) | 804 (6.2%) | 0.074 | 700 (6.6%) | 690 (6.5%) | 0.003 |
| Benzodiazepines | 2,817 (10.5%) | 1,121 (8.7%) | 0.062 | 988 (9.3%) | 965 (9.1%) | 0.007 |
| COPD And Asthma Meds | 6,943 (25.9%) | 2,758 (21.4%) | 0.107 | 2,371 (22.3%) | 2,348 (22.1%) | 0.004 |
| Corticosteroids Oral | 7,502 (28.0%) | 3,180 (24.7%) | 0.076 | 2,701 (25.4%) | 2,669 (25.2%) | 0.006 |
| Dementia Meds | 283 (1.1%) | 249 (1.9%) | 0.072 | 163 (1.5%) | 162 (1.5%) | 0.001 |
| Gabapentinoids | 4,883 (18.2%) | 1,901 (14.8%) | 0.094 | 1,653 (15.6%) | 1,635 (15.4%) | 0.004 |
| Nitrates & Other Antianginal Meds | 1,720 (6.4%) | 752 (5.8%) | 0.024 | 625 (5.9%) | 619 (5.8%) | 0.002 |
| NSAIDs | 8,158 (30.5%) | 3,401 (26.4%) | 0.09 | 2,899 (27.3%) | 2,879 (27.1%) | 0.003 |
| Opioids | 7,433 (27.8%) | 2,994 (23.2%) | 0.104 | 2,609 (24.5%) | 2,590 (24.4%) | 0.003 |
| Statins | 19,952 (74.5%) | 9,458 (73.4%) | 0.025 | 7,872 (74.1%) | 7,828 (73.8%) | 0.006 |
| Other Lipid Lowering Agents | 4,275 (16.0%) | 1,890 (14.7%) | 0.036 | 1,586 (14.9%) | 1,572 (14.8%) | 0.003 |
| UTI Antibiotics | 4,401 (16.4%) | 1,899 (14.7%) | 0.047 | 1,612 (15.2%) | 1,595 (15.0%) | 0.004 |
| ***Healthcare resource utilization*** |  |  |  |  |  |  |
| Internist visits, mean(SD) | 5.4(7.11) | 4.5(5.59) | 0.135 | 4.6(5.92) | 4.6(5.75) | 0.001 |
| Cardiologist visits, mean(SD) | 1.3(3.16) | 1.1(2.99) | 0.043 | 1.1(2.96) | 1.1(3.02) | 0.002 |
| Endocrinologist visits, mean(SD) | 0.8(1.98) | 0.5(1.33) | 0.218 | 0.5(1.25) | 0.5(1.41) | 0.007 |
| Nephrologist visits, mean(SD) | 0.1(0.70) | 0.1(0.59) | 0.028 | 0.1(0.63) | 0.1(0.59) | 0 |
| N creatinine, mean(SD) | 0.1(0.54) | 0.1(0.48) | 0.05 | 0.1(0.49) | 0.1(0.51) | 0.003 |
| N hba1c test, mean(SD) | 2.6(2.27) | 2.1(1.85) | 0.239 | 2.2(1.93) | 2.2(1.93) | 0.006 |
| N microalbuminuria, mean(SD) | 1.0(1.23) | 0.8(0.99) | 0.176 | 0.8(1.07) | 0.8(1.02) | 0.012 |
| Number of emergency department visits, mean(SD) | 0.5(1.54) | 0.4(1.10) | 0.076 | 0.4(1.18) | 0.4(1.15) | 0.009 |
| Number of hospital visits, mean(SD) | 0.1(0.42) | 0.1(0.40) | 0.031 | 0.1(0.39) | 0.1(0.40) | 0.001 |
| Metabolic Or Renal Panel Orders | 19,961 (74.6%) | 9,749 (75.7%) | 0.026 | 7,994 (75.2%) | 7,964 (75.1%) | 0.003 |
| Cardiovascular stress test | 1,530 (5.7%) | 699 (5.4%) | 0.013 | 570 (5.4%) | 576 (5.4%) | 0.003 |
| Flu Vaccine | 5,418 (20.2%) | 2,877 (22.3%) | 0.051 | 2,325 (21.9%) | 2,316 (21.8%) | 0.001 |
| ***Laboratory results**** |  |  |  |  |  |  |
| HbA1c, mean(SD) | 9.0(1.93) | 8.9(1.85) | 0.062 | 9.1(1.92) | 8.9(1.83) | 0.108 |
| Creatinine, mean(SD) | 1.0(0.39) | 1.0(0.30) | 0 | 1.0(0.35) | 1.0(0.30) | 0.005 |
| eGFR, mean(SD) | 85.2(22.01) | 83.1(21.58) | 0.096 | 83.8(21.52) | 83.2(21.73) | 0.028 |
| HDL cholesterol, mean(SD) | 43.8(12.23) | 44.9(12.54) | 0.09 | 44.3(12.22) | 44.7(12.32) | 0.032 |
| LDL cholesterol, mean(SD) | 92.0(36.63) | 91.3(35.94) | 0.021 | 92.2(37.08) | 91.4(35.83) | 0.022 |
| Total cholesterol, mean(SD) | 173.8(48.89) | 175.0(47.46) | 0.026 | 174.9(48.96) | 174.9(47.50) | 0.002 |
| Triglyceride, mean(SD) | 185.5(91.23) | 180.7(90.20) | 0.053 | 184.6(91.44) | 182.7(91.29) | 0.014 |

Abbreviations: ACEI: Angiotensin-Converting Enzyme Inhibitor; AKI: Acute Kidney Injury; ARB: Angiotensin II Receptor Blocker; CBVD: Cerebrovascular Disease; CKD: Chronic Kidney Disease; COPD: Chronic Obstructive Pulmonary Disease; CVD: Cardiovascular Disease; DKA: Diabetic Ketoacidosis; DPP-4i: Dipeptidyl Peptidase-4 inhibitor; ECG: Electrocardiogram; ED: Emergency Department; eGFR: Estimated Glomerular Filtration Rate; HbA1c: Hemoglobin A1c; HDL: High-Density Lipoprotein; HONK: Hyperosmolar Non-Ketotic State; LDL: Low-Density Lipoprotein; MASLD: Metabolic-dysfunction Associated Steatotic Liver Disease; MI: Myocardial Infarction; MRA: Mineralocorticoid Receptor Antagonist; NSAID: Non-Steroidal Anti-Inflammatory Drug; PA: Physician Assistant; PAD: Peripheral Artery Disease; SD: Standard Deviation; SES: Socioeconomic Status; TZD: Thiazolidinedione; UTI: Urinary Tract Infection.

*Available in 13% of the population.

**Appendix Table 5. Baseline characteristics of GLP-1RA initiators who added empagliflozin or sulfonylureas**

| **Characteristic** | | **Empagliflozin** | **Sulfonylureas** | **SMD** | **Empagliflozin** | **Sulfonylureas** | **SMD** |
| --- | --- | --- | --- | --- | --- | --- | --- |
|  |  | **N=31,073** | **N=26,558** |  | **N=19,727** | **N=19,683** |  |
| Age, mean(SD) | | 58.0(12.16) | 61.3(12.51) | 0.267 | 59.1(12.34) | 59.1(12.34) | 0 |
| Sex, F | | 14,374 (46.3%) | 13,706 (51.6%) | 0.107 | 9,811 (49.7%) | 9,777 (49.7%) | 0.001 |
| Race | |  |  |  |  |  |  |
| White | | 11,742 (66.4%) | 12,170 (70.9%) | 0.097 | 7,920 (68.2%) | 7,890 (68.2%) | 0.001 |
| Black | | 2,172 (12.3%) | 1,915 (11.2%) | 0.035 | 1,361 (11.7%) | 1,359 (11.7%) | 0.001 |
| Asian | | 515 (2.9%) | 457 (2.7%) | 0.015 | 323 (2.8%) | 328 (2.8%) | 0.003 |
| Hispanic | | 2,184 (12.3%) | 1,697 (9.9%) | 0.078 | 1,337 (11.5%) | 1,332 (11.5%) | 0 |
| Other/Unknown | | 1,076 (6.1%) | 927 (5.4%) | 0.029 | 668 (5.8%) | 669 (5.8%) | 0.001 |
| Year of cohort entry | |  |  |  |  |  |  |
| 2014 | | 58 (0.2%) | 460 (1.7%) | 0.159 | 48 (0.2%) | 237 (1.2%) | 0.113 |
| 2015 | | 514 (1.7%) | 1,850 (7.0%) | 0.264 | 460 (2.3%) | 916 (4.7%) | 0.127 |
| 2016 | | 1,330 (4.3%) | 2,576 (9.7%) | 0.214 | 1,156 (5.9%) | 1,311 (6.7%) | 0.033 |
| 2017 | | 2,841 (9.1%) | 3,185 (12.0%) | 0.093 | 2,453 (12.4%) | 1,634 (8.3%) | 0.136 |
| 2018 | | 3,297 (10.6%) | 3,686 (13.9%) | 0.1 | 2,474 (12.5%) | 2,473 (12.6%) | 0.001 |
| 2019 | | 6,214 (20.0%) | 4,396 (16.6%) | 0.089 | 3,748 (19.0%) | 3,739 (19.0%) | 0 |
| 2020 | | 6,801 (21.9%) | 4,799 (18.1%) | 0.096 | 4,158 (21.1%) | 4,120 (20.9%) | 0.004 |
| 2021 | | 5,469 (17.6%) | 3,269 (12.3%) | 0.149 | 3,012 (15.3%) | 3,017 (15.3%) | 0.002 |
| 2022 | | 4,549 (14.6%) | 2,337 (8.8%) | 0.182 | 2,218 (11.2%) | 2,235 (11.4%) | 0.004 |
| ***Burden of comorbidity/deprivation*** | |  |  |  |  |  |  |
| Combined comorbidity score, mean(SD) | | 1.7(2.21) | 1.8(2.35) | 0.022 | 1.7(2.22) | 1.7(2.23) | 0.001 |
| Frailty score percent, mean(SD) | | 15.4(4.58) | 15.7(4.57) | 0.047 | 15.4(4.51) | 15.4(4.50) | 0 |
| SES index score, mean(SD) | | 57.9(7.25) | 57.4(7.06) | 0.082 | 57.7(7.18) | 57.7(7.18) | 0 |
| ***Cardiovacular comorbidities*** | |  |  |  |  |  |  |
| Baseline CVD | | 9,930 (32.0%) | 8,254 (31.1%) | 0.019 | 5,850 (29.7%) | 5,755 (29.2%) | 0.009 |
| Heart Failure (before cohort entry) | | 2,737 (8.8%) | 2,284 (8.6%) | 0.007 | 1,578 (8.0%) | 1,572 (8.0%) | 0 |
| Heart Failure (between cohort entry and augmentation) | | 1,724 (5.5%) | 1,237 (4.7%) | 0.04 | 891 (4.5%) | 882 (4.5%) | 0.002 |
| Unstable angina (before cohort entry) | | 760 (2.4%) | 578 (2.2%) | 0.018 | 410 (2.1%) | 426 (2.2%) | 0.006 |
| Unstable angina (between cohort entry and augmentation) | | 413 (1.3%) | 236 (0.9%) | 0.042 | 207 (1.1%) | 182 (0.9%) | 0.013 |
| Stable angina (before cohort entry) | | 6,419 (20.7%) | 5,252 (19.8%) | 0.022 | 3,708 (18.8%) | 3,691 (18.8%) | 0.001 |
| Stable angina (between cohort entry and date of augmentation) | | 4,079 (13.1%) | 2,831 (10.7%) | 0.076 | 2,108 (10.7%) | 2,092 (10.6%) | 0.002 |
| Acute MI (before cohort entry) | | 760 (2.4%) | 498 (1.9%) | 0.039 | 355 (1.8%) | 391 (2.0%) | 0.014 |
| Acute MI (between cohort entry and augmentation) | | 414 (1.3%) | 186 (0.7%) | 0.063 | 179 (0.9%) | 153 (0.8%) | 0.014 |
| Old MI/sequalae (before cohort entry) | | 1,263 (4.1%) | 944 (3.6%) | 0.027 | 696 (3.5%) | 687 (3.5%) | 0.002 |
| Old MI/sequalae (between cohort entry and date of augmentation) | | 709 (2.3%) | 433 (1.6%) | 0.047 | 328 (1.7%) | 330 (1.7%) | 0.001 |
| Stroke (before cohort entry) | | 2,024 (6.5%) | 1,820 (6.9%) | 0.014 | 1,229 (6.2%) | 1,220 (6.2%) | 0.001 |
| Stroke (between cohort entry and date of augmentation) | | 1,138 (3.7%) | 873 (3.3%) | 0.02 | 633 (3.2%) | 622 (3.2%) | 0.003 |
| Other cerebrovascular diseases | | 434 (1.4%) | 401 (1.5%) | 0.009 | 274 (1.4%) | 274 (1.4%) | 0 |
| History Coronary Procedure | | 2,303 (7.4%) | 1,681 (6.3%) | 0.043 | 1,223 (6.2%) | 1,211 (6.2%) | 0.002 |
| History Coronary Procedure | | 1,326 (4.3%) | 846 (3.2%) | 0.057 | 646 (3.3%) | 635 (3.2%) | 0.003 |
| Late Effects Of CBVD | | 726 (2.3%) | 692 (2.6%) | 0.017 | 454 (2.3%) | 464 (2.4%) | 0.004 |
| PAD | | 1,343 (4.3%) | 1,323 (5.0%) | 0.031 | 862 (4.4%) | 857 (4.4%) | 0.001 |
| ***Diabetes and other comorbidities*** | |  |  |  |  |  |  |
| Diabetic Nephropathy | | 4,039 (13.0%) | 3,886 (14.6%) | 0.047 | 2,565 (13.0%) | 2,558 (13.0%) | 0 |
| Diabetic Neuropathy | | 6,250 (20.1%) | 5,379 (20.3%) | 0.003 | 3,807 (19.3%) | 3,803 (19.3%) | 0.001 |
| Diabetic Retinopathy | | 1,272 (4.1%) | 1,122 (4.2%) | 0.007 | 746 (3.8%) | 744 (3.8%) | 0 |
| Diabetes Other Complications | | 2,809 (9.0%) | 2,165 (8.2%) | 0.032 | 1,636 (8.3%) | 1,627 (8.3%) | 0.001 |
| Diabetes Other Ophthalmic Complications | | 595 (1.9%) | 699 (2.6%) | 0.048 | 391 (2.0%) | 414 (2.1%) | 0.008 |
| Diabetes Peripheral Circulation | | 2,249 (7.2%) | 1,828 (6.9%) | 0.014 | 1,300 (6.6%) | 1,295 (6.6%) | 0 |
| Diabetic Foot | | 688 (2.2%) | 592 (2.2%) | 0.001 | 405 (2.1%) | 407 (2.1%) | 0.001 |
| Valve Disorders | | 1,493 (4.8%) | 1,298 (4.9%) | 0.004 | 873 (4.4%) | 874 (4.4%) | 0.001 |
| Other CVD | | 2,671 (8.6%) | 2,282 (8.6%) | 0 | 1,566 (7.9%) | 1,569 (8.0%) | 0.001 |
| Other Cardiac Dysrhythmia | | 2,106 (6.8%) | 1,893 (7.1%) | 0.014 | 1,300 (6.6%) | 1,297 (6.6%) | 0 |
| Atrial Fibrillation | | 1,710 (5.5%) | 1,650 (6.2%) | 0.03 | 1,071 (5.4%) | 1,069 (5.4%) | 0 |
| Cardiac Conduction Disorders | | 785 (2.5%) | 659 (2.5%) | 0.003 | 444 (2.2%) | 448 (2.3%) | 0.002 |
| Cardiomyopathy | | 733 (2.4%) | 554 (2.1%) | 0.019 | 393 (2.0%) | 391 (2.0%) | 0 |
| AKI | | 707 (2.3%) | 774 (2.9%) | 0.04 | 444 (2.3%) | 446 (2.3%) | 0.001 |
| CKD stages 1-2 | | 881 (2.8%) | 698 (2.6%) | 0.013 | 518 (2.6%) | 516 (2.6%) | 0 |
| CKD stages 3-4 | | 1,689 (5.4%) | 2,557 (9.6%) | 0.159 | 1,298 (6.6%) | 1,282 (6.5%) | 0.003 |
| CKD unspecified | | 787 (2.5%) | 1,042 (3.9%) | 0.079 | 564 (2.9%) | 560 (2.8%) | 0.001 |
| Diabetes without complications | | 23,558 (75.8%) | 20,991 (79.0%) | 0.077 | 15,132 (76.7%) | 15,185 (77.1%) | 0.01 |
| MASLD | | 1,590 (5.1%) | 1,095 (4.1%) | 0.047 | 909 (4.6%) | 898 (4.6%) | 0.002 |
| All Cancer | | 1,607 (5.2%) | 1,686 (6.3%) | 0.051 | 1,097 (5.6%) | 1,092 (5.5%) | 0.001 |
| Anxiety | | 3,758 (12.1%) | 3,035 (11.4%) | 0.021 | 2,322 (11.8%) | 2,326 (11.8%) | 0.001 |
| Asthma | | 1,968 (6.3%) | 1,769 (6.7%) | 0.013 | 1,274 (6.5%) | 1,278 (6.5%) | 0.001 |
| COPD | | 1,919 (6.2%) | 1,865 (7.0%) | 0.034 | 1,256 (6.4%) | 1,249 (6.3%) | 0.001 |
| Dementia | | 391 (1.3%) | 510 (1.9%) | 0.053 | 290 (1.5%) | 294 (1.5%) | 0.002 |
| Depression | | 4,145 (13.3%) | 3,516 (13.2%) | 0.003 | 2,616 (13.3%) | 2,620 (13.3%) | 0.001 |
| Disorders Ca Phos Mg Balance | | 856 (2.8%) | 823 (3.1%) | 0.02 | 536 (2.7%) | 529 (2.7%) | 0.002 |
| Disorders Electrolyte Balance | | 1,420 (4.6%) | 1,416 (5.3%) | 0.035 | 896 (4.5%) | 883 (4.5%) | 0.003 |
| Disorders Of Fluid | | 613 (2.0%) | 592 (2.2%) | 0.018 | 385 (2.0%) | 387 (2.0%) | 0.001 |
| Dorsopathies | | 6,795 (21.9%) | 6,228 (23.5%) | 0.038 | 4,402 (22.3%) | 4,402 (22.4%) | 0.001 |
| Edema | | 1,829 (5.9%) | 1,771 (6.7%) | 0.032 | 1,169 (5.9%) | 1,157 (5.9%) | 0.002 |
| Erectile Dysfunction | | 1,066 (3.4%) | 832 (3.1%) | 0.017 | 641 (3.3%) | 638 (3.2%) | 0.001 |
| Genital Infection | | 963 (3.1%) | 955 (3.6%) | 0.028 | 620 (3.1%) | 746 (3.8%) | 0.035 |
| GERD | | 4,202 (13.5%) | 3,822 (14.4%) | 0.025 | 2,681 (13.6%) | 2,701 (13.7%) | 0.004 |
| Hyperglycemia | | 17,229 (55.4%) | 12,604 (47.5%) | 0.16 | 10,046 (50.9%) | 10,046 (51.0%) | 0.002 |
| Hyperlipidemia | | 22,640 (72.9%) | 19,242 (72.5%) | 0.009 | 14,168 (71.8%) | 14,131 (71.8%) | 0.001 |
| Hypertension | | 23,393 (75.3%) | 20,480 (77.1%) | 0.043 | 14,812 (75.1%) | 14,778 (75.1%) | 0 |
| Hypertensive Nephropathy | | 1,522 (4.9%) | 1,804 (6.8%) | 0.081 | 1,034 (5.2%) | 1,023 (5.2%) | 0.002 |
| Hyperthyroidism | | 214 (0.7%) | 194 (0.7%) | 0.005 | 137 (0.7%) | 145 (0.7%) | 0.005 |
| Hypoglycemia | | 3,697 (11.9%) | 2,537 (9.6%) | 0.076 | 2,060 (10.4%) | 2,049 (10.4%) | 0.001 |
| Hypothyroidism | | 4,825 (15.5%) | 4,470 (16.8%) | 0.035 | 3,175 (16.1%) | 3,154 (16.0%) | 0.002 |
| Hypovolemia | | 537 (1.7%) | 525 (2.0%) | 0.018 | 345 (1.7%) | 340 (1.7%) | 0.002 |
| Liver Disease | | 503 (1.6%) | 418 (1.6%) | 0.004 | 296 (1.5%) | 299 (1.5%) | 0.001 |
| Lower Limb Amputations | | 199 (0.6%) | 153 (0.6%) | 0.008 | 111 (0.6%) | 108 (0.5%) | 0.002 |
| Lower limb infections | | 866 (2.8%) | 819 (3.1%) | 0.018 | 553 (2.8%) | 552 (2.8%) | 0 |
| HONK/DKA | | 389 (1.3%) | 293 (1.1%) | 0.014 | 214 (1.1%) | 215 (1.1%) | 0.001 |
| Kidney and urinary stones | | 791 (2.5%) | 761 (2.9%) | 0.02 | 504 (2.6%) | 523 (2.7%) | 0.006 |
| Fracture | | 89 (0.3%) | 120 (0.5%) | 0.027 | 64 (0.3%) | 71 (0.4%) | 0.006 |
| Osteoporosis | | 931 (3.0%) | 1,036 (3.9%) | 0.05 | 666 (3.4%) | 665 (3.4%) | 0 |
| Major Bleeding | | 1,683 (5.4%) | 1,492 (5.6%) | 0.009 | 1,067 (5.4%) | 1,070 (5.4%) | 0.001 |
| Metabolic Or Renal Panel | | 23,001 (74.0%) | 20,068 (75.6%) | 0.035 | 14,594 (74.0%) | 14,554 (73.9%) | 0.001 |
| Obstructive Sleep Apnea | | 5,979 (19.2%) | 4,255 (16.0%) | 0.085 | 3,359 (17.0%) | 3,367 (17.1%) | 0.002 |
| Orthostatic Hypotension Or Synoc | | 615 (2.0%) | 593 (2.2%) | 0.018 | 385 (2.0%) | 417 (2.1%) | 0.012 |
| Osteoarthritis | | 4,265 (13.7%) | 4,158 (15.7%) | 0.055 | 2,810 (14.2%) | 2,806 (14.3%) | 0 |
| Pneumonia | | 692 (2.2%) | 628 (2.4%) | 0.009 | 429 (2.2%) | 420 (2.1%) | 0.003 |
| Proteinuria | | 1,374 (4.4%) | 1,098 (4.1%) | 0.014 | 796 (4.0%) | 794 (4.0%) | 0 |
| Sleep Disorders | | 2,300 (7.4%) | 1,945 (7.3%) | 0.003 | 1,416 (7.2%) | 1,415 (7.2%) | 0 |
| UTI | | 1,928 (6.2%) | 2,244 (8.4%) | 0.086 | 1,363 (6.9%) | 1,438 (7.3%) | 0.015 |
| ***Behavioral risk factors*** | |  |  |  |  |  |  |
| Smoking | | 5,051 (16.3%) | 4,146 (15.6%) | 0.018 | 3,075 (15.6%) | 3,074 (15.6%) | 0.001 |
| Alcohol Abuse Dependence | | 228 (0.7%) | 191 (0.7%) | 0.002 | 140 (0.7%) | 143 (0.7%) | 0.002 |
| Drug Abuse Dependence | | 436 (1.4%) | 342 (1.3%) | 0.01 | 263 (1.3%) | 262 (1.3%) | 0 |
| Overweight | | 1,975 (6.4%) | 1,622 (6.1%) | 0.01 | 1,229 (6.2%) | 1,230 (6.3%) | 0.001 |
| Morbid Obesity | | 8,011 (25.8%) | 5,359 (20.2%) | 0.133 | 4,404 (22.3%) | 4,404 (22.4%) | 0.001 |
| Normal Under Wt | | 418 (1.3%) | 445 (1.7%) | 0.027 | 299 (1.5%) | 302 (1.5%) | 0.002 |
| Obesity | | 7,456 (24.0%) | 5,309 (20.0%) | 0.097 | 4,220 (21.4%) | 4,193 (21.3%) | 0.002 |
| Unspecified Obesity | | 8,948 (28.8%) | 6,312 (23.8%) | 0.114 | 4,993 (25.3%) | 4,971 (25.3%) | 0.001 |
| ***History of treatment*** | |  |  |  |  |  |  |
| Days of background therapy, mean(SD) | 215.0(314.28) | | 154.3(242.98) | 0.216 | 182.4(278.52) | 166.9(260.84) | 0.058 |
| ***Concomitant medications*** | |  |  |  |  |  |  |
| No. glucose-lowering medications | |  |  |  |  |  |  |
| 1 | | 22,253 (71.6%) | 20,997 (79.1%) | 0.173 | 15,231 (77.2%) | 15,186 (77.2%) | 0.001 |
| 2 | | 7,731 (24.9%) | 4,890 (18.4%) | 0.158 | 3,945 (20.0%) | 3,952 (20.1%) | 0.002 |
| 3+ | | 1,089 (3.5%) | 671 (2.5%) | 0.057 | 551 (2.8%) | 545 (2.8%) | 0.002 |
| Metformin | | 24,171 (77.8%) | 19,428 (73.2%) | 0.108 | 14,893 (75.5%) | 14,869 (75.5%) | 0.001 |
| Insulin | | 13,301 (42.8%) | 7,253 (27.3%) | 0.329 | 6,198 (31.4%) | 6,178 (31.4%) | 0.001 |
| Long Term Insulin Use | | 7,128 (22.9%) | 3,916 (14.7%) | 0.211 | 3,311 (16.8%) | 3,291 (16.7%) | 0.002 |
| DPP-4i | | 7,722 (24.9%) | 7,076 (26.6%) | 0.041 | 5,053 (25.6%) | 5,020 (25.5%) | 0.003 |
| TZD | | 2,294 (7.4%) | 2,437 (9.2%) | 0.065 | 1,645 (8.3%) | 1,640 (8.3%) | 0 |
| No. cardiovascular medications | |  |  |  |  |  |  |
| 0 | | 7,952 (25.6%) | 6,545 (24.6%) | 0.022 | 5,198 (26.4%) | 5,177 (26.3%) | 0.001 |
| 1 | | 8,658 (27.9%) | 7,543 (28.4%) | 0.012 | 5,555 (28.2%) | 5,551 (28.2%) | 0.001 |
| 2 | | 7,802 (25.1%) | 6,825 (25.7%) | 0.014 | 4,961 (25.1%) | 4,941 (25.1%) | 0.001 |
| 3 | | 4,707 (15.1%) | 4,091 (15.4%) | 0.007 | 2,908 (14.7%) | 2,904 (14.8%) | 0 |
| 4+ | | 1,954 (6.3%) | 1,554 (5.9%) | 0.018 | 1,105 (5.6%) | 1,111 (5.6%) | 0.002 |
| Antiarrhythmics | | 497 (1.6%) | 384 (1.4%) | 0.013 | 259 (1.3%) | 260 (1.3%) | 0.001 |
| Anticoagulants Injectables | | 151 (0.5%) | 142 (0.5%) | 0.007 | 88 (0.4%) | 99 (0.5%) | 0.008 |
| Anticoagulants Oral | | 2,211 (7.1%) | 1,939 (7.3%) | 0.007 | 1,332 (6.8%) | 1,318 (6.7%) | 0.002 |
| Anticonvulsants | | 6,616 (21.3%) | 5,537 (20.8%) | 0.011 | 4,048 (20.5%) | 4,035 (20.5%) | 0.001 |
| Antidepressants | | 10,169 (32.7%) | 8,198 (30.9%) | 0.04 | 6,208 (31.5%) | 6,204 (31.5%) | 0.001 |
| Antiparkinsonian Meds | | 930 (3.0%) | 887 (3.3%) | 0.02 | 627 (3.2%) | 628 (3.2%) | 0.001 |
| Antiplatelet Agents | | 3,542 (11.4%) | 2,567 (9.7%) | 0.056 | 1,907 (9.7%) | 1,901 (9.7%) | 0 |
| Antipsychotics | | 1,065 (3.4%) | 873 (3.3%) | 0.008 | 679 (3.4%) | 669 (3.4%) | 0.002 |
| Anxiolytics Hypnotics | | 2,702 (8.7%) | 2,134 (8.0%) | 0.024 | 1,622 (8.2%) | 1,610 (8.2%) | 0.002 |
| Benzodiazepines | | 3,455 (11.1%) | 3,082 (11.6%) | 0.015 | 2,194 (11.1%) | 2,191 (11.1%) | 0 |
| Beta Blockers | | 11,266 (36.3%) | 9,442 (35.6%) | 0.015 | 6,728 (34.1%) | 6,702 (34.0%) | 0.001 |
| Calcium Channel Blockers | | 8,142 (26.2%) | 7,266 (27.4%) | 0.026 | 5,141 (26.1%) | 5,126 (26.0%) | 0 |
| Loop Diuretics | | 3,947 (12.7%) | 3,514 (13.2%) | 0.016 | 2,362 (12.0%) | 2,357 (12.0%) | 0 |
| Thiazide | | 9,476 (30.5%) | 8,254 (31.1%) | 0.013 | 5,994 (30.4%) | 5,995 (30.5%) | 0.002 |
| MRA Potassium Sparing Diuretics | | 1,618 (5.2%) | 1,113 (4.2%) | 0.048 | 845 (4.3%) | 842 (4.3%) | 0 |
| COPD And Asthma Meds | | 8,602 (27.7%) | 6,818 (25.7%) | 0.045 | 5,173 (26.2%) | 5,168 (26.3%) | 0.001 |
| Corticosteroids Oral | | 9,320 (30.0%) | 7,457 (28.1%) | 0.042 | 5,598 (28.4%) | 5,584 (28.4%) | 0 |
| Dementia Meds | | 311 (1.0%) | 422 (1.6%) | 0.052 | 239 (1.2%) | 246 (1.3%) | 0.003 |
| Gabapentinoids | | 5,746 (18.5%) | 4,877 (18.4%) | 0.003 | 3,524 (17.9%) | 3,515 (17.9%) | 0 |
| Histamine Blockers | | 1,585 (5.1%) | 1,349 (5.1%) | 0.001 | 981 (5.0%) | 963 (4.9%) | 0.004 |
| Nitrates & Other Antianginal Meds | | 1,963 (6.3%) | 1,369 (5.2%) | 0.05 | 1,019 (5.2%) | 1,018 (5.2%) | 0 |
| NSAIDs | | 9,847 (31.7%) | 7,682 (28.9%) | 0.06 | 5,889 (29.9%) | 5,871 (29.8%) | 0.001 |
| Opioids | | 9,312 (30.0%) | 7,888 (29.7%) | 0.006 | 5,694 (28.9%) | 5,681 (28.9%) | 0 |
| Other Antihypertensive Meds | | 1,687 (5.4%) | 1,662 (6.3%) | 0.035 | 1,091 (5.5%) | 1,088 (5.5%) | 0 |
| Statins | | 22,799 (73.4%) | 18,283 (68.8%) | 0.1 | 13,780 (69.9%) | 13,761 (69.9%) | 0.001 |
| Other Lipid Lowering Agents | | 5,042 (16.2%) | 3,638 (13.7%) | 0.071 | 2,831 (14.4%) | 2,813 (14.3%) | 0.002 |
| UTI Antibiotics | | 5,507 (17.7%) | 5,231 (19.7%) | 0.051 | 3,591 (18.2%) | 3,574 (18.2%) | 0.001 |
| ***Healthcare resource utilization*** | |  |  |  |  |  |  |
| Internist visits, mean(SD) | | 6.0(8.02) | 5.5(7.34) | 0.069 | 5.6(7.42) | 5.6(7.59) | 0.006 |
| Cardiologist visits, mean(SD) | | 1.3(3.48) | 1.1(2.82) | 0.084 | 1.1(3.00) | 1.1(2.84) | 0.007 |
| Endocrinologist visits, mean(SD) | | 1.0(2.24) | 0.7(1.76) | 0.16 | 0.7(1.88) | 0.7(1.88) | 0.003 |
| Nephrologist visits, mean(SD) | | 0.1(0.90) | 0.2(1.01) | 0.048 | 0.1(0.89) | 0.1(0.89) | 0.001 |
| No pa visits, mean(SD) | | 1.3(3.35) | 1.2(3.06) | 0.047 | 1.2(3.12) | 1.2(3.13) | 0.021 |
| N creatinine, mean(SD) | | 0.1(0.57) | 0.1(0.51) | 0.016 | 0.1(0.53) | 0.1(0.50) | 0.006 |
| N hba1c test, mean(SD) | | 2.9(2.55) | 2.4(2.10) | 0.19 | 2.6(2.28) | 2.5(2.21) | 0.019 |
| N microalbuminuria, mean(SD) | | 1.1(1.32) | 0.9(1.15) | 0.162 | 0.9(1.18) | 0.9(1.20) | 0.002 |
| Number of emergency department visits, mean(SD) | | 0.5(1.56) | 0.5(1.44) | 0.028 | 0.5(1.35) | 0.5(1.51) | 0.003 |
| Number of hospital visits, mean(SD) | | 0.1(0.47) | 0.1(0.48) | 0.008 | 0.1(0.44) | 0.1(0.45) | 0.004 |
| ***Diagnostic and preventive screening services*** | |  |  |  |  |  |  |
| Cardiovascular stress test | | 1,624 (5.2%) | 1,225 (4.6%) | 0.028 | 907 (4.6%) | 905 (4.6%) | 0 |
| ECG And Other Cardiac Imaging | | 3,834 (12.3%) | 2,993 (11.3%) | 0.033 | 2,149 (10.9%) | 2,133 (10.8%) | 0.002 |
| Continuous Glucose Monitoring | | 1,049 (3.4%) | 452 (1.7%) | 0.107 | 403 (2.0%) | 394 (2.0%) | 0.003 |
| Lipid Test Order | | 22,153 (71.3%) | 18,422 (69.4%) | 0.042 | 13,944 (70.7%) | 13,764 (69.9%) | 0.017 |
| Flu Vaccine | | 6,458 (20.8%) | 6,015 (22.6%) | 0.045 | 4,184 (21.2%) | 4,183 (21.2%) | 0.001 |
| Pneumococcal Vaccine | | 2,067 (6.7%) | 1,918 (7.2%) | 0.022 | 1,361 (6.9%) | 1,290 (6.6%) | 0.014 |
| ***Lab results**** | |  |  |  |  |  |  |
| HbA1c, mean(SD) | | 8.9(1.90) | 8.9(2.19) | 0.042 | 8.8(1.90) | 9.0(2.26) | 0.091 |
| Creatinine, mean(SD) | | 0.9(0.33) | 1.0(0.45) | 0.135 | 0.9(0.33) | 1.0(0.46) | 0.086 |
| eGFR, mean (SD) | | 86.6(21.99) | 81.5(23.87) | 0.221 | 85.0(22.13) | 83.4(23.22) | 0.068 |
| HDL cholesterol, mean(SD) | | 43.5(11.98) | 44.7(14.00) | 0.091 | 44.2(12.12) | 44.3(14.23) | 0.009 |
| LDL cholesterol, mean(SD) | | 91.9(36.74) | 92.4(39.60) | 0.013 | 93.0(36.70) | 92.5(40.31) | 0.014 |
| Total cholesterol, mean(SD) | | 172.4(47.31) | 174.4(54.01) | 0.039 | 174.2(46.59) | 174.1(55.81) | 0.002 |
| Triglyceride, mean(SD) | | 189.0(101.73) | 188.0(97.68) | 0.010 | 188.7(101.21) | 189.2(98.58) | 0.005 |

Abbreviations: ACEI: Angiotensin-Converting Enzyme Inhibitor; AKI: Acute Kidney Injury; ARB: Angiotensin II Receptor Blocker; CBVD: Cerebrovascular Disease; CKD: Chronic Kidney Disease; COPD: Chronic Obstructive Pulmonary Disease; CVD: Cardiovascular Disease; DKA: Diabetic Ketoacidosis; DPP-4i: Dipeptidyl Peptidase-4 inhibitor; ECG: Electrocardiogram; ED: Emergency Department; eGFR: Estimated Glomerular Filtration Rate; HbA1c: Hemoglobin A1c; HDL: High-Density Lipoprotein; HONK: Hyperosmolar Non-Ketotic State; LDL: Low-Density Lipoprotein; MASLD: Metabolic-dysfunction Associated Steatotic Liver Disease; MI: Myocardial Infarction; MRA: Mineralocorticoid Receptor Antagonist; NSAID: Non-Steroidal Anti-Inflammatory Drug; PA: Physician Assistant; PAD: Peripheral Artery Disease; SD: Standard Deviation; SES: Socioeconomic Status; TZD: Thiazolidinedione; UTI: Urinary Tract Infection.

*Available in 13% of the population.

**Appendix Table 6. Follow-up time and censoring reasons after applying matching weights**

|  | **Empagliflozin + GLP-1RA** | **Empagliflozin + DPP-4i** |
| --- | --- | --- |
| **Modified MACE** |  |  |
| Follow-up in days (median, IQR) | 171 (90, 366) | 167 (94, 333) |
| Follow-up in days (mean, SD) | 287 (295) | 270 (273) |
| **Censoring reasons (%)** |  |  |
| Bariatric surgery | 0.27 | 0.23 |
| End of data availability | 22.92 | 20.39 |
| End of enrollment | 16.92 | 16.14 |
| Background drug discontinuation | 25.89 | 30.68 |
| Augmented drug discontinuation | 27.93 | 17.97 |
| Outcome | 1.44 | 1.69 |
| Switching between GLP-1RA and DPP-4i | 2.07 | 10.65 |
| Switching from empagliflozin to other SGLT2i | 2.01 | 2.25 |
| Weight loss drugs initiation (tirzepatide, saxenda, wegovy) | 0.56 | 0 |
| **HHF or mortality** |  |  |
| Follow-up in days (median, IQR) | 172 (91, 369) | 168 (95, 334) |
| Follow-up in days (mean, SD) | 289 (297) | 271 (274) |
| **Censoring reasons** |  |  |
| Bariatric surgery | 0.27 | 0.23 |
| End of data availability | 23.13 | 20.43 |
| End of enrollment | 17.09 | 16.17 |
| Background drug discontinuation | 26.02 | 30.86 |
| Augmented drug discontinuation | 27.98 | 18.07 |
| Outcome | 0.84 | 1.31 |
| Switching between GLP-1RA and DPP-4i | 2.1 | 10.66 |
| Switching from empagliflozin to other SGLT2i | 2.01 | 2.28 |
| Weight loss drugs initiation (tirzepatide, saxenda, wegovy) | 0.56 | 0 |
|  | **GLP-1RA + empagliflozin** | **GLP-1RA +**  **Sulfonylureas** |
| **Modified MACE** |  |  |
| Follow-up in days (median, IQR) | 132 (71, 292) | 111 (61, 228) |
| Follow-up in days (mean, SD) | 233 (275) | 189 (225) |
| **Censoring reasons** |  |  |
| Bariatric surgery | 0.32 | 0.27 |
| End of data availability | 19.1 | 14.4 |
| End of enrollment | 14.56 | 11.73 |
| Background drug discontinuation | 38.71 | 43.57 |
| Augmented drug discontinuation | 20.55 | 21.29 |
| Outcome | 0.9 | 1.08 |
| Switching between SGLT-2 and Sulfonylureas | 3.6 | 7.53 |
| Switching from empagliflozin to other SGLT2i | 1.93 | 0 |
| Weight loss drugs initiation (tirzepatide, saxenda, wegovy) | 0.34 | 0.13 |
| **HHF or mortality** |  |  |
| Follow-up in days (median, IQR) | 132 (71, 293) | 111 (61, 228) |
| Follow-up in days (mean, SD) | 234 (276) | 189 (226) |
| **Censoring reasons** |  |  |
| Bariatric surgery | 0.32 | 0.27 |
| End of data availability | 19.17 | 14.45 |
| End of enrollment | 14.58 | 11.79 |
| Background drug discontinuation | 38.74 | 43.54 |
| Augmented drug discontinuation | 20.61 | 21.38 |
| Outcome | 0.71 | 0.9 |
| Switching between SGLT-2 and Sulfonylureas | 3.62 | 7.54 |
| Switching from empagliflozin to other SGLT2i | 1.92 | 0 |
| Weight loss drugs initiation (tirzepatide, saxenda, wegovy) | 0.34 | 0.13 |

Abbreviations: DPP-4i: Dipeptidyl Peptidase-4 Inhibitor; GLP-1RA: Glucagon-Like Peptide-1 Receptor Agonist; HHF: Hospitalization for Heart Failure; IQR: Interquartile Range; MACE: Major Adverse Cardiovascular Events; SD: Standard Deviation; SGLT2i: Sodium–Glucose Cotransporter-2 Inhibitor.

**Appendix Figure 1. Study design scheme for combination therapies initiating empagliflozin first**

Abbreviations: DPP-4i: Dipeptidyl Peptidase-4 Inhibitor; ESKD: End-Stage Kidney Disease; GLP-1RA: Glucagon-Like Peptide-1 Receptor Agonist; SGLT2i: Sodium–Glucose Cotransporter-2 Inhibitor; SU: Sulfonylurea; T2D: Type 2 Diabetes.

**Appendix Figure 2. Study design scheme for combination therapies initiating GLP-1RA first**

Abbreviations: DPP-4i: Dipeptidyl Peptidase-4 Inhibitor; ESKD: End-Stage Kidney Disease; GLP-1RA: Glucagon-Like Peptide-1 Receptor Agonist; SGLT2i: Sodium–Glucose Cotransporter-2 Inhibitor; SU: Sulfonylurea; T2D: Type 2 Diabetes.

**Appendix Figure 3. Flow chart Empagliflozin background**

|  | **CMS** | **MKT** | **CDM** |
| --- | --- | --- | --- |
| Had empagliflozin RX after a 6-month washout period | 256,977 | 292,089 | 286,366 |
| No prevalent use of SGLT-2, GLP-1RA, or DPP-4 in a 6-month washout | 139,512 | 164,072 | 169,774 |
| Had date of augmentation with GLP-1RA or DPP-4i | 20,552 | 31,371 | 28,688 |
| No ambiguous age or sex information, age >= 65 (>=18 in commercial claims) | 20,487 | 31,365 | 28,685 |
| At least one baseline diagnosis code for type 2 diabetes | 20,288 | 29,542 | 27,452 |
| **Excluded with:** |  |  |  |
| - End-staged kidney disease | 181 | 84 | 155 |
| - HIV | 83 | 176 | 157 |
| - Nursing home visits | 1,119 | 216 | 1,070 |
| - Transplant | 105 | 84 | 90 |
| - Type 1 diabetes | 1,430 | 1,351 | 1,311 |
| - bariatric | 29 | 38 | 38 |
| - cirrhosis | 324 | 226 | 335 |
| - gestational diabetes | 1 | 24 | 16 |
| - hepatitis | 27 | 21 | 37 |
| - pancreatitis | 168 | 196 | 197 |
| - secondary diabetes | 822 | 566 | 735 |
| **Remains** | **16,710** | **26,849** | **23,857** |
| **Excluded with:** |  |  |  |
| - more than 1 SGLT-2 agent on first cohort entry date | 16 | 18 | 16 |
| - more than 1 GLP-1RA or DPP-4i agent on second cohort entry date | 47 | 57 | 47 |
| **Remains** | **16,647** | **26,774** | **23,795** |
| After tirzepatide, Saxenda, Wegovy exclusion | 16,647 | 26,774 | 23,795 |
| Augmenters only and date of augmentation before censor date | 9,013 | 16,183 | 14,463 |
| **Main analysis cohort**: follow-up begins before censor date | **8,300** | **14,868** | **13,240** |

Abbreviations: CDM: Optum’s De-identified Clinformatics Data Mart Database; CMS: Centers for Medicare and Medicaid Services; DPP-4i: Dipeptidyl Peptidase-4 Inhibitor; GLP-1RA: Glucagon-Like Peptide-1 Receptor Agonist; HIV: Human Immunodeficiency Virus; MKT: IBM MarketScan Commercial Claims Database; RX: Prescription; SGLT2i: Sodium–Glucose Cotransporter-2 Inhibitor.

**Appendix Figure 4. Flow chart: GLP-1RA background**

|  | **CMS** | **MKT** | **CDM** |
| --- | --- | --- | --- |
| Had GLP-1R (except Saxenda or Wegovy or tirzepatide) RX after a 6-month washout period | 397,067 | 554,325 | 403,044 |
| No SGLT-2, GLP-1RA, or SU Rxs in a 6-month washout period | 213,759 | 357,612 | 245,158 |
| Have second entry date (60 days discontinuation) | 20,047 | 28,522 | 25,170 |
| No ambiguous age or sex information, age >= 65 | 19,957 | 28,505 | 25,162 |
| At least one baseline diagnosis code for type 2 diabetes | 19,846 | 27,051 | 23,985 |
| **Excluded with:** |  |  |  |
| - End-staged kidney disease | 332 | 129 | 189 |
| - HIV | 72 | 145 | 166 |
| - Nursing home visits | 1,394 | 212 | 155 |
| - Transplant | 132 | 104 | 96 |
| - Type 1 diabetes | 2,148 | 1,649 | 1,488 |
| - bariatric | 42 | 46 | 20 |
| - cirrhosis | 333 | 230 | 342 |
| - gestational diabetes | 1 | 38 | 19 |
| - hepatitis | 37 | 19 | 36 |
| - pancreatitis | 208 | 207 | 187 |
| - secondary diabetes | 1,015 | 586 | 646 |
| **Remains** | **15,285** | **24,089** | **21,045** |
| **Excluded with:** |  |  |  |
| - more than 1 GLP-1RA agent on first cohort entry date | 6 | 10 | 7 |
| - initiate both empagliflozin and SU agent on second cohort entry date | 56 | 128 | 112 |
| - initiate SGLT2i agents (other than empagliflozin) on second cohort entry date | 4 | 12 | 11 |
| - initiate more than 1 Sulfonylureas on second cohort entry date | 12 | 46 | 10 |
| **Remains** | **15,207** | **23,893** | **20,905** |
| After tirzepatide, Saxenda, Wegovy exclusion | 15,207 | 23,842 | 20,896 |
| Augmenters only and date of augmentation before censor date | 8,878 | 15,778 | 13,660 |

Abbreviations: CDM: Optum’s De-identified Clinformatics Data Mart Database; CMS: Centers for Medicare and Medicaid Services; DPP-4i: Dipeptidyl Peptidase-4 Inhibitor; GLP-1RA: Glucagon-Like Peptide-1 Receptor Agonist; HIV: Human Immunodeficiency Virus; MKT: IBM MarketScan Commercial Claims Database; RX: Prescription; SGLT2i: Sodium–Glucose Cotransporter-2 Inhibitor.

**Appendix Figure 5. Observed and bias-adjusted relative risks demonstrating the impact of strong unmeasured confounding by frailty and socioeconomic status**

| a. Estimates adjusted for frailty (RRobs=0.61) | b. Estimates adjusted for SES (RRobs=0.61) |
| --- | --- |
|  |  |
| c. Estimates adjusted for frailty (RRobs=0.68) | d. Estimates adjusted for SES (RRobs=0.68) |
|  |  |

*Here, we examined the impact of unmeasured confounding on the extreme protective associations observed in our study. This range of scenarios is based on strong negative confounding by frailty and SES, with strong imbalance between our exposure and comparators.

**Confounding by frailty**

*Methods*: In this sensitivity analysis, we explored the effect of potential strong unmeasured confounding by frailty.

We assumed:

- Frailty is a strong risk factor that doubles or triples cardiovascular disease risk (RR_CD_ up to 3.25), AND
- This risk factor must be 2.5 times less common in our treated group (20% vs 50%),

which means 20% of empagliflozin-GLP-1RA combination therapies and 50% of the comparator combination therapies was assumed to have frailty. Based on literature, the effect of frailty on MACE and HHF could range from 1.7-2.5 (**plausible range**), as reported in James K, Jamil Y, Kumar M, Kwak MJ, Nanna MG, Qazi S, Troy AL, Butt JH, Damluji AA, Forman DE, Orkaby AR. Frailty and Cardiovascular Health. Journal of the American Heart Association 2024;13:e031736).

*Results*: For the observed RR of 0.61, the most extreme assumption of strongest effect of frailty on CVD risk (RR_CD_ of 3.25) led to the bias-adjusted RR of 0.89. However, over the **plausible ranges** of RR_CD_ of 1.7-2.5, the bias-adjusted RR ranged from **0.73 to 0.82**. For the observed RR of 0.68, over the plausible ranges of RR_CD_ of 1.7-2.5, the bias-adjusted RR ranged from 0.81 to 0.92.

**Confounding by socioeconomic status (SES)**

*Methods*: We explored the effect of potential strong unmeasured confounding by SES (social deprivation index), which was coded as deciles (the higher the decile, the greater the affluence),

- Social deprivation index is a strong risk factor: for every decile increase in social deprivation index, i.e., lower SES, doubles the cardiovascular disease risk (RR_CD_ up to 0.5), AND
- Our treated group has higher SES (top 3^rd^ vs. 8^th^ decile),

We note that the plausible RR_CD_ based on the literature was 0.7. This is based on the reported study in literature (Aryee EK, Ndumele CE, Khan SS, Crews D, Sang Y, Grams M, Ballew S, Matsushita K, Coresh J. Abstract 13270: Social Deprivation Index and Cardiovascular Disease Risk Among 2.5 Million Patients in the US. Circulation 2023;148:A13270-A13270).

*Results*:

For the observed RR of 0.61, when the RR_CD_ 0.7 (a plausible value based on literature), the bias-adjusted RR was 0.73. More extreme assumptions led to the bias-adjusted RR of 0.86. For the observed RR of 0.68, over the plausible value of RR_CD_ of 0.7, the bias-adjusted RR was 0.81. Only the extreme assumption of twice the higher CVD risk for every decile increase in the social deprivation index led to the bias-adjusted RR of 0.96.

**For both confounders, the E-value to explain away the observed relative risk of 0.61 (95%CI: 0.48, 0.77) was 2.66, and for 0.68 (95%CI: 0.56, 0.81), the E-value was 2.3.**

Abbreviations: CI: Confidence Interval; CVD: Cardiovascular Disease; E-value: Evidence Value; HHF: Hospitalization for Heart Failure; MACE: Major Adverse Cardiovascular Events; RR: Relative Risk; RRadj: Bias-Adjusted Relative Risk; RRobs: Observed Relative Risk; RRcd: Relative Risk Due to Confounding; SES: Socioeconomic Status.
